# Ready-to-Use Therapeutic Food for Catch-Up Growth in Children after an Episode of *Plasmodium falciparum* Malaria: An Open Randomised Controlled Trial

**DOI:** 10.1371/journal.pone.0035006

**Published:** 2012-04-25

**Authors:** Saskia van der Kam, Todd Swarthout, Oscar Niragira, Alyson Froud, Eric Mukomena Sompwe, Clair Mills, Stephanie Roll, Peter Tinnemann, Leslie Shanks

**Affiliations:** 1 Médecins Sans Frontières, Amsterdam, The Netherlands; 2 Médecins Sans Frontières, Lubumbashi, Democratic Republic of Congo; 3 Ministère de Santé, Division Province du Katanga, Lubumbashi, Democratic Republic of Congo; 4 University of Auckland, Auckland, New Zealand; 5 Institute for Social Medicine, Epidemiology and Health Economics, Charité University Medical Centre, Berlin, Germany; The George Washington University Medical Center, United States of America

## Abstract

**Background:**

Catch-up growth after an infection is essential for children to maintain good nutritional status. To prevent malnutrition, WHO recommends that children are given one additional healthy meal per day during the 2 weeks after onset of illness. We investigated to what extent ready-to-use therapeutic food (RUTF) promotes catch-up growth in children after an acute, uncomplicated episode of *Plasmodium falciparum* malaria.

**Methods:**

We did an open randomised trial of children aged 6–59 months with confirmed malaria who attended a Médecins Sans Frontières-supported outpatient clinic in Katanga Province, Democratic Republic of Congo. All children received a clinical examination and malaria treatment. Patients were then randomly assigned to either an RUTF group, who received daily supplemental RUTF (a high-protein peanut-based paste) for 14 days, or to a control group, who received no supplemental food. Children were weighed at baseline and on days 14 and 28. The primary outcome was mean weight change after 14 days' RUTF. Analysis was by intention-to-treat.

**Results:**

93 children received RUTF and 87 received no food supplementation. At day 14, the RUTF group had a mean weight gain of 353 g compared with 189 g in the control group (difference 164 [95%CI 52–277], p = 0.005). However, at day 28 there was no statistically significant difference between the groups (539 g versus 414 g, respectively [p = 0.053]). Similarly, rate of weight gain per kg bodyweight per day was significantly higher at day 14 in the RUTF group (2.4 g/kg per day versus 1.3 g/kg per day, p = 0.005) but at day 28 was 1.9 g/kg per day in the RUTF group versus 1.5 g/kg per day in the control group (p = 0.076).

**Conclusions:**

Children receiving RUTF for 14 days after effective treatment of an uncomplicated malaria episode had a faster weight gain than children not given supplementation, reducing the period that children were at risk of malnutrition.

**Trial Registration:**

ClinicalTrials.gov NCT00819858

## Introduction

The causes of malnutrition are multifactorial, involving not only an inadequate diet but also recurrent infections [1], [2]. Young children have an especially high susceptibility to acute infectious diseases such as malaria, diarrhoea, and acute respiratory infections, which are generally associated with weight loss [3]. This occurs because infections can decrease food intake due to poor appetite, impair nutrient absorption, cause direct nutrient losses by diarrhoea or vomiting, increase metabolic requirements or catabolic losses [4], and impair transport of nutrients to target tissues. If disease-associated weight loss is not prevented or compensated for, it can lead to longer-term effects on nutrition [2], [5]. This weight loss, in turn, increases susceptibility to further infections, perpetuating a cycle towards a further reduced nutritional state [3], [5]–[8].

Effects of short-term weight loss during episodes of acute infection can be mitigated by good nutrition, allowing for successful convalescence and adequate catch-up growth [9], [10]. To prevent malnutrition, WHO recommends that children are given one additional healthy meal daily during the 2 weeks after the onset of illness [11]. However, in many developing countries this strategy is unlikely to be effective, since the ingredients necessary for a healthy meal are often not available. Therefore, a more effective strategy in resource-poor areas could be to provide nutrient-rich high-quality food supplements for ill children. Other than a study in Gambia [10], there has been little evidence to support the effectiveness of this approach. In that study, weight loss in children with acute diarrhoea was effectively treated by providing a supplement enriched with vitamins and minerals for 2 weeks [10]. In the group that did not receive supplementation, no significant overall weight gain occurred. This finding raised the question of whether supplementation with a nutrient-rich food would also be effective for convalescence after infections other than acute diarrhoea, such as malaria.

The following hypothesis was therefore tested: children aged 6–59 months with confirmed P falciparum malaria who are provided with a RUTF supplement (Plumpy'nut) of 500 kcal per day for 14 days will show significantly better weight gain after 14 and 28 days compared to a similar patient group not provided with RUTF.

Plumpy'nut (Nutriset, Malaunay, France) is an industrially produced high-quality nutritional supplement that has been shown to promote weight gain in malnourished children [12]. The treatment is an individually packed lipid based paste made up of peanuts, milk powder, sugar, oil, and fortified with micronutrients. Although the supplement contains iron and folic acid, no adverse effects have been reported on either the incidence or severity of malaria [12].

Malaria kills more than a million people annually, with children under 5 years of age being the most vulnerable. More than 90% of malaria deaths occur in Africa, where around two-thirds of the population are at high risk of infection [13]. We assessed the effect of a 14-day distribution of ready-to-use therapeutic food (RUTF) on weight gain in children after an episode of acute uncomplicated *Plasmodium falciparum* malaria. This study was a pilot to guide further research on the effect of supplementation in various diseases and settings.

## Methods

The protocol for this trial and supporting CONSORT checklist are available as supporting information; see Checklist S1 and Protocol S1.

### Study location and participants

This study took place in the outpatient department (OPD) of the Centre de Santé de Référence Mutabi–a referral hospital supported by Médecins Sans Frontières (MSF)–in Dubié, Katanga Province, southern Democratic Republic of Congo. Dubié is situated in a forest region. There is no defined hunger season, with people able to cultivate maize, sorghum, cassava, beans, leafy greens and fruits throughout the year. In addition, fish and poultry products are regularly available. Though this can be interrupted by periods of conflict, forcing the population to flee into the forest and thus abandoning their crops, the area experienced relative calm before and during the study period. The study was conducted just after harvesting of mangos, a rich source of vitamins. Breastfeeding is commonly practiced, although foods of limited nutritional value (incl. water-based porridges) are introduced as early as 3 months of age. In addition, children weaned from breastfeeding often do not receive milk and other high quality foods.

**Figure 1:**
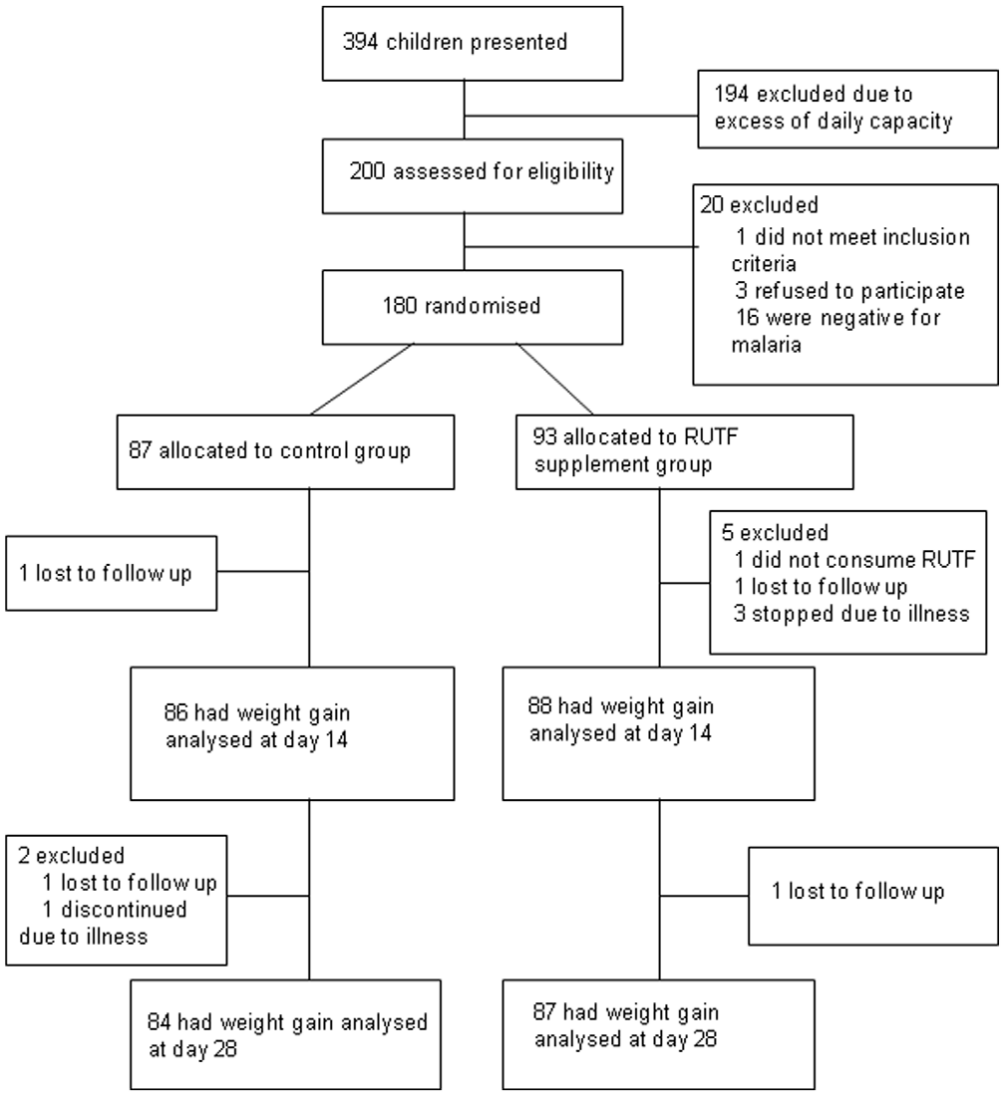
Trial profile.

The major childhood diseases are diarrhea, respiratory tract infections and malaria. Malaria is endemic throughout year with peak periods between September and March. In 2007, 54 671 children under 5 years of age consulted the outpatient department, of whom 21 939 (40%) were treated for uncomplicated malaria. Amongst total malaria cases seen in the OPD, approximately half occur in children under 5 years of age.

**Table 1:** Baseline characteristics.

	RUTF	Control
Number	93	87
Female^a^	36 (38.7%)	44 (50.6%)
Age (months)^b^	29.6 (14.5)	28.0 (14.7)
Age ≤36 months^a^	56 (60.2%)	54 (62.0%)
Breastfeeding, partial a,d	21 (22.6%)	32 (36.8%)
Days of illness before visit^c^	2.8 (2, 2–3)	3.2 (3, 2–3)
Fevera,e	86 (92.5%)	82 (94.3%)
Parasite density^b^	37 414 (59 302)	37 089 (52 317)
Cougha,e,f	48 (51.6%)	47 (54.0%)
Diarrhoeaa,e, g	5 (5.4%)	6 (6.9%)
Vomitinga,e, h	5 (5.4%)	2 (2.3%)
Appetite less than normala,e	77 (82.8%)	60 (69.0%)
Weight (kg) b	10.24 (2.40)	10.20 (2.53)
Weight-for-height z-score^b^	−0.53 (1.00)	−0.46 (0.99)
Weight-for-height z-score <−2^a^	6 (6.8%)	5 (5.8%)
MUAC (mm)^b^	149 (13)	151 (11)
Weight-for-age z-score^b^	−1.91 (1.10)	−1.72 (1.18)
Height-for-age z-score^b^	−2.67 (1.46)	−2.48 (1.46)
Mother is caretaker^a^	80 (86.0%)	77 (88.5%)
Age (years)^b^	28.9 (9.9)	29.1 (8.2)
No education^a^	27 (29.0%)	20 (23.0%)
More than 4 years primary school level^a^	42 (46.7%)	39 (45.2%)
Occupation agriculture^a^	78 (88.6%)	69 (80.2%)
Partner's occupation agriculture^a^	51 (57.9%)	45 (52.3%)
Household wealth score^c^	1.7 (2, 1–3)	1.7 (2, 1–3)
Less than two meals previous day^a^	25 (26.9%)	33 (37.9%)
Number of meals previous day^c^	1.69 (2, 1–2)	1.73 (2, 1–2)

RUTF: ready-to-use therapeutic food. MUAC: middle-upper arm circumference.

a Number (%).

b mean ± SD: standard deviation.

c mean (median, IQR: interquartile range).

d 91 patients analysed in the intervention group and 83 in the control group.

symptom in the 14 days prior to study inclusion.

f axillary temperature >38°C.

g 3 or more liquid stools in any 24 hour period.

h occasional persistent vomiting.

Inclusion criteria included children age 6–59 months, presenting at the OPD, confirmed uncomplicated *Plasmodium falciparum* malaria, and voluntary informed written consent from the caretaker. Exclusion criteria included exclusive breastfeeding, severe malnourishment (middle-upper arm circumference of less than 110 mm and/or bilateral oedema or a weight-for-height *z*-score [WHZ] of less than –3 [WHO 2005 reference]; signs of severe malaria as defined by WHO guidelines, a known history of allergy to malaria drugs, a sibling enrolled in the study, living further than 30 minutes walking distance from the clinic, and severe illness or needing hospitalisation. Patients were withdrawn if they met any of the exclusion criteria during the study, or if they failed to complete the malaria treatment schedule. Informed consent was obtained by reading the consent form aloud in French or the appropriate local language.

**Table 2:** Mean weight gain (g).

	RUTF	Control	Difference	p-value
Day 0–14a,b	352.7 (275.4–430.1) 365.0	188.6 (105.7–271.5) 386.8	164.1 (51.6–276.6)	0.005
Day 14–28a,c	187.1 (125.9–248.3) 287.1	228.6 (166.6–290.5) 285.2	−41.4 (−127.9–250.7)	0.345
Day 0–28a,c	539.3 (447.8–630.8) 428.2	414.3 (325.9–502.7) 407.5	125.0 (−1.4–251.4)	0.053

a mean (95% CI) SD.

b RUTF n = 88, control n = 86.

c RUTF n = 87, control n = 84.

### Study procedures

At point of entry into the OPD clinic, all children with suspected uncomplicated malaria (defined as having a fever greater than 38.0°C, a reported history of fever within the past 48 hours, or both), were screened using the *P. falciparum* rapid diagnostic test (RDT), Paracheck (Orchid Biomedical System, Goa, India), by the routine non-study clinical staff. Children were then seen by a non-study clinician for routine clinical examination and treatment. Children positive for malaria by RDT were referred and accompanied to the study clinic, located in the same compound.

**Table 3:** Mean rate of weight gain (g/kg per day).

	RUTF	Control	Difference	p-value
Day 0–14a,b	2.36 (1.85–2.87) 2.40	1.29 (0.75–1.84) 2.53	1.07 (0.33–1.81)	0.005
Day 14–28a,c	1.37 (0.90–1.82) 2.12	1.70 (1.28–2.12) 1.95	−0.33 (−0.95–0.29)	0.293
Day 0–28a,c	1.87 (1.55–2.18) 1.47	1.48 (1.19–1.77) 1.35	0.389 (−0.04–0.81)	0.076

a mean (95% CI) SD.

b RUTF n = 88, control n = 86.

b RUTF n = 87, control n = 84.

The number of children presenting at the OPD clinic with suspected malaria regularly exceeded the study team's daily screening capacity. Therefore, depending on the daily estimation of patients attending the OPD clinic, the study coordinator systematically used a selection interval of every second or third child arriving at the study clinic to screen for study inclusion. To minimise risk of selection bias, OPD staff were not informed of the day's selection interval. Thick blood film microscopy was used as the gold standard to confirm infection with *P falciparum* malaria. Children who tested negative by microscopy were excluded from the study, and those who were positive were further screened for inclusion.

**Table 4:** Number gaining any weight (>0 g).

	RUTF	Control	Odds ratio^b^	p-value
Day 0–14a,c	74 (84.1)	61 (70.9)	2.17 (1.04–4.52)	0.040
Day 14–28a,d	64 (73.6)	67 (79.8)	0.71 (0.35–1.44)	0.340
Day 0–28a,d	79 (90.8)	77 (91.7)	0.90 (0.31–2.60)	0.842

a n (%).

b OR (95% CI).

c RUTF n = 88, control n = 86.

d RUTF n = 87, control n = 84.

All recruited children were randomly assigned to either an RUTF group (hereafter referred to as RUTF group) receiving RUTF (500 kcal per day for 14 days) or to a control group without food supplementation. 200 numbered pieces of paper of equal size and weight were placed into a cloth bag, and a number was drawn out by the study coordinator in front of the caretaker and child. All even numbers were allocated to the RUTF group and odd numbers allocated to the control group. Once the number was allocated it was removed. This method was chosen to reduce the risk of misunderstanding or suspicion in a setting where the study population would likely not have understood the logic of a computer generated allocation scheme. With the use of standard labelled RUTF packets, blinding of participants and clinic staff was not practical. Staff performing weight measurements and laboratory testing were blind to study group allocation.

**Table 5:** Mean rate of weight change (g/kg per day), stratified by sex.

		RUTF a	Control^a^	Difference^b^	p value¥
Day 0–14	Female	2.83 (1.92–3.75) 34	1.03 (0.16–1.90) 44	1.80 (0.54–3.06)	
	Male	2.06 (1.46–2.67) 54	1.57 (0.90–2.22) 42	0.49 (0.39–1.38)	0.163
Day 0–28	Female	2.15 (1.62–2.67) 34	1.39 (0.95–1.82) 44	0.76 (0.09–1.43)	
	Male	1.68 (1.29–2.08 ) 53	1.58 (1.18–1.99 ) 40	0.10 (−0.46–0.67)	0.215

a Mean (95% CI) n.

b Mean (95% CI).

¥ likelihood ratio test (LRT) for effect modification.

**Table 6:** Mean rate of weight change (g/kg per day), stratified by breastfeeding.

	Breastfeeding	RUTF a	Control a	Difference b	p value¥
Day 0–14	yes	1.42 (0.35–2.50) 20	1.12 (0.43–1.82) 32	0.30 (0.89–1.49)	
	no	2.64 (2.06–3.21) 68	1.39 (0.61–2.17) 54	1.24 (0.31–2.18)	0.230
Day 0–28	yes	1.60 (0.89–2.33, 20	1.40 (0.99–1.82) 30	0.19 (−0.57–0.95)	
	no	1.95 (1.60–2.30) 67	1.52 (1.12–1.92) 54	0.43 (0.10–0.95)	0.490

a Mean (95% CI) n.

b Mean (95% CI).

¥ likelihood ratio test (LRT) for effect modification.

Upon inclusion, all children received malaria treatment as per local MoH/MSF protocol (artesunate 4 mg/kg for 3 days plus amodiaquine 10 mg/kg a day for 3 days). Malaria treatment was directly supervised (DOTS) at the study clinic for the three consecutive days. Microscopy was repeated on the third day to confirm parasite clearance. Those patients remaining slide-positive on the third day were asked to return each day until parasite clearance was microscopically confirmed or treatment failure was established.

At study inclusion, anthropometric data were collected and a detailed questionnaire was completed to obtain background and clinical information. The RUTF group received 14 sachets of RUTF, with the first sachet opened in the clinic and instructions given to the caretaker to guide proper use. Adherence to the food supplement was measured in three ways: the caretakers were asked how many sachets the child had consumed, how many days the sachets were consumed, and to bring back the empty sachets to the clinic. Weight measurements were taken at inclusion (day 0), day 14, and day 28, using an electronic weighing scale (SECA model 835, Seca Corporation, Hamburg, Germany). A detailed questionnaire and medical exam were completed on day 14 and day 28. To minimise differences in weight within the day, routine follow-up visits and weighing activities were scheduled for the same time of day as the first visit, and the caretakers were reminded by a home visitor a day before their scheduled appointment.

Caretakers were encouraged to bring their children to the study clinic at any time if they had questions or felt that they needed medical care. In line with WHO recommendations, all caretakers were informed about the importance of giving sick children an additional healthy meal per day for 14 days. All participants who completed the study received an insecticide impregnated bednet and soap to compensate for participation.

The primary outcome was weight change (g) from day 0 to day 14. Secondary outcomes included: weight change from day 0 to 28, and from day 14 to 28; rate of weight gain (g/kg per day) at day 14 and 28; the proportion of children with any weight gain, and contribution of other factors to convalescence after an episode of malaria. The study questionnaire for each clinical visit captured key health indicators for the two weeks prior to clinical visit, including cough, fever, diarrhoea, vomiting and appetite. A household wealth score was calculated using the possession of specified household items (incl. bicycle, watch, radio, and generator) and livestock.

Various measures ensured the quality of the data collected, including patient files kept on site for future visits; establishing a pattern of patient flow to ensure efficiency and reduce risk of contamination; continuous monitoring and training of staff; data entry on the same evening of the patient visit, allowing for clarification the following day; double data entry after completion of the study.

### Laboratory

Blood smears for microscopic detection of parasites were stained with 10% pre-filtered Giemsa. Asexual parasites were counted against 200–500 leucocytes and converted to number of parasites per volume assuming 8,000 leucocytes/μL blood. Slides were considered negative when no parasites were detected after viewing 100 microscopic fields. Microscopists, unaware of study allocation, read all slides. Internal quality control included a blind second reading of a proportion of the slides, as per MSF protocols [14]. External quality control consisted of a random sample of 20% blood slides sent to an external reference laboratory (Path Care, Nairobi Kenya).

### Statistical analysis

Parameters for the sample size calculation were, in part, based on the result of a previous supplementation study of children with diarrhoea [10]. We extrapolated the evidence that nutritional supplementation would address weight gain related to a malaria infection in a manner similar to that seen by Hoare and colleagues for diarrhoeal disease [10].

Parameters used for initial sample size calculations included a power of 80%, a 5% level of significance for a two-sided t-test, and an assumed mean weight gain after 14 days of 0.09 kg (SD 0.44) in the control group and 0.44 kg (SD 0.33) in the RUTF group [10]. This resulted in a sample size of 24 participants per study group (STATA software, version 8). With no clear data on weight change amongst malaria cases, and using 24 participants per study group as a minimum, we worked to define the highest number of participants logistically feasible to enrol during the available four week period. This resulted in 100 per group (including 20 additional children for potential lost to follow up and withdrawals from the study).

**Table 7:** Children having a symptom in the 2 weeks before day 0, at day 14, and at day 28.

		Fever^c^	Cough	Diarrhoea^d^	Appetite < normal
**Day 0**	RUTF a	86 (92.5%)	48 (51.6%)	5 (5.4%)	77 (82.8%)
	Control a	82 (94.3%)	47 (54.0%)	6 (6.9%)	60 (69.0%)
	OR b	0.75 (0.23–2.45)	0.91 (0.50–1.63)	0.77 (0.22–2.61)	2.16 (1.07–4.38)
	p-value	0.630	0.746	0.671	0.032
**Day 14**	RUTF a	5 (5.7%)	16 (18.2%)	4 (4.6%)	4 (4.5%)
	Control a	8 (9.3%)	32 (37.2%)	7 (8.1%)	12 (14.0%)
	OR b	0.59 (0.18–1.87)	0.38 (0.19–0.75)	0.54 (0.15–1.91)	0.30 (0.09–0.950)
	p-value	0.368	0.006	0.336	0.041
**Day 28**	RUTF a	6 (6.9%)	10 (11.5%)	4 (4.6%)	3 (3.5%)
	Control a	11 (13.1%)	12 (14.3%)	4 (4.8%)	4 (4.8%)
	OR b	0.49 (0.17–1.40)	0.78 (0.32–1.91)	0.96 (0.23–3.98)	0.71 (0.15–3.29)
	p-value	0.182	0.590	0.959	0.666

Vomiting 8 cases in total: not reported.

a Number (%).

b OR (95% CI).

c axillary temperature >38°C.

c Described as 3 or more liquid stools in any 24 hour period.

Statistical analysis was completed based on a parallel RCT design with an allocation ratio of 1∶1 and an intention to treat principle. To verify the randomization assumption, we compared the prevalence of baseline characteristics between the two study groups. Graphical methods were used to test for normality of baseline and outcome variables, including histograms and box plots. Mean weight changes and other continuous variables were compared by two-sample t-test (two-sided), and categorical data were analysed using Pearson's chi-squared test with continuity correction or Fisher's exact test if frequencies were small. Median and interquartile range (IQR) were reported for discrete data. Presence of outliers and their impact were assessed. Statistical analysis was completed using STATA version 8.2. The likelihood ratio test (LRT) was used to assess effect modification, recognising that tests for effect modification are known to have limited statistical power. The statistician was not blinded to allocation.

A limited number of variables were investigated for effect modification, including household socioeconomic status; maternal breastfeeding practices; child's age, sex, and nutritional status. We estimated adjusted means and odds ratios from generalized linear mixed-effects models with weight gain as the outcome. Predictors included household socioeconomic status, maternal age and education, child's age and sex, breastfeeding practices, appetite, nutritional status, and duration (days) of malaria clinical symptoms before seeking treatment. Controlling for identified and *a priori* potential confounders showed no statistical, clinical, or operational difference in results. As such, we report only unadjusted results.

## Results

Between January 6 and February 16, 2009, 394 children with suspected malaria were referred to the study clinic. Of these, 200 were screened for eligibility, including microscopy for confirmation of active malaria infection; the 194 who were not screened received a medical exam and appropriate treatment. During the screening process, twenty children were excluded: one did not meet the inclusion criteria, three chose to not participate after the being informed through the consent procedure, and 16 had blood slide results that were negative for malaria.

The 180 participants who were recruited were randomly assigned by the study coordinator to either the control group (n = 87) or the RUTF group (n = 93; Figure 1). The study was stopped early due to unexpected field constraints related to changes in security. This resulted in a total recruitment meeting the minimum sample size of 180 children and an unequal distribution of children per study group.

Quality control on the 20% of malaria microscopy slides checked by the reference laboratory revealed an agreement of 97.5%. Discrepancies in microscopy would not have impacted participant inclusion or treatment decisions.

At 14 days, all patients in the RUTF group were reported to have eaten ten or more of the fourteen RUTF sachets received at inclusion 82 (93.2%) caretakers in the RUTF group reported that their child had eaten all 14 RUTF sachets, with 76 (84.4%) returning all empty sachets. During return visits, all but three participants were weighed at the same time of day as previously (within a 3 hour margin). The three participants who presented outside these time limits returned the day after their appointment at the right time; the weight gain rate was calculated over this period.

The average age of the children was 28.8 (SD 14.6) months, and was similar for both groups. 44.4% (80) of participants were girls. Through verifying the randomisation assumption for distribution of baseline characteristics, three variables were noteworthy without being statistically significant. These include a higher percentage of girls in the control group (50.6%, 38.7%), more reported vomiting in the RUTF group (5.4%, 2.3%) and a greater proportion of households in the control group eating less than two meals during the previous day (37.9%, 26.9%). An additional two variables showed statistically significant difference at baseline, including more children in the control group being partially breastfed (36.8%, 22.6%; p = 0.03) and more children in the RUTF group having a less than normal appetite (82.8%, 69.0%; p = 0.03). Controlling for these potential confounders showed no statistical, clinical, or operational difference. For this reason, we chose not to adjust for potential confounders. The nutrition indicators were similar for both groups, with a total mean WHZ <−0.50 (WHO 2005 reference). 11 (6%) children had a WHZ below-2 (WHO 2005 reference). The mean weight-for-age z-score was-1.82 and height-for-age z-score was-2.58 (WHO 2005 reference; Table 1).

The child was most often accompanied by the mother to the clinic, with other relations including grandparents, fathers, and siblings (Table 1). Caretaker socio-demographics and household wealth scores did not differ significantly by group, with the two study groups having an equal median and IQR (median: 2, IQR: 1–3). At inclusion, the reported number of meals eaten per household during the day before the interview ranged between 0 and 3 meals, with the study groups having equal median and IQR (median: 2, IQR: 1–2). 122 (67.7%) caretakers reportedly consumed 2–3 meals. Only one household in the RUTF group reportedly ate no meal during the day before the interview.

Nine children were withdrawn or lost to follow-up: six from the RUTF group and three from the control group. One participant in the RUTF group was lost to follow-up on day 14 (reason unknown), and one at day 28 when the caretaker went with the child to the lake to fish. Four participants in the RUTF group were withdrawn: one because the caretaker reported that the child did not receive the supplement (child was healthy with appetite), two due to admission to hospital, and one due to death. A thorough case evaluation of the death revealed that the cause was not related to study activities. In the control group, one patient was lost to follow-up on day 14 and one on day 28 because of agriculture and fishing activities. On day 20, one child was withdrawn due to illness and subsequent admission to hospital (Figure 1). There were no malaria treatment failures.

### Weight gain

At day 14, mean weight gain was significantly higher in the RUTF group (352.7 *vs* 188.6 g in the control group; difference 164.1 g [95% CI 51.6–276.6], p = 0.005; Table 2). Mean weight gain at day 28 was still higher in the RUTF group, with a difference approaching statistical significance (difference 125.0 g [95% CI-1.4 to 251.4], p = 0.053).

The rate of weight gain was also significantly higher in the RUTF group (2.36 *vs* 1.29 g/kg per day p = 0.005) during the supplementation period (days 0–14), although it was slightly higher in the control group during days 14–28 (1.37 *vs* 1.07 g/kg per day, p = 0.293). After 28 days, the RUTF group showed a higher overall rate of weight gain (1.87 *vs* 1.48 g/kg per day), with a difference approaching statistical significance (p = 0.076; Table 3).

A higher proportion of children in the RUTF group gained any weight (i.e. weight gain>0 g) than in the control group at day 14 (84.1% *vs* 70.9%; odds ratio [OR] 2.17, 95% CI 1.04–4.52; p = 0.040). However, during the 14-day follow up after the supplementation ended, a smaller proportion of children in the RUTF group gained weight compared with the control group (73.6% *vs* 79.8%; OR 0.71, 95% CI 0.35–1.44; p = 0.340), although this was not significant. Within the total 28 day study period, an almost equal proportion of children gained any weight in the RUTF and control groups (90.8% *vs* 91.7%; OR 0.90, 95% CI 0.31–2.60; p = 0.842; Table 4).

Gender and breastfeeding were identified as effect modifiers. Though likelihood ratio tests (LRT) did not show statistical significance, results at day 14 showed girls in the RUTF group with a much higher rate of weight gain than the males (1.80 g/kg/day (95%CI: 0.54–3.06) vs 0.49 g/kg/day (95%CI: 0.39 to 1.38), respectively). A notable difference was also recorded after 28 days of follow up. The rate of weight gain at day 14 was also much higher amongst children in the RUTF group who were not breastfed compared to children partially breastfed (1.24 g/kg/day (95%CI: 0.31–2.18) *vs* 0.30 g/kg/day (95%CI: 0.89–1.49) respectively). A similar difference was also recorded after 28 days of follow up. (Tables 5 and 6).

Amongst those reporting reduced appetite at inclusion, the rate of weight gain at day 14 was 2.46 g/kg per day in the RUTF group and 1.11 g/kg per day in the control group (p = 0.001), whereas children in the two groups reporting normal or better appetite during the same time period had similar rates of weight gain: 1.88 g/kg per day in the RUTF group and 1.76 g/kg per day in the control group (p = 0.843).

As shown in Table 7, there were no significant differences between study groups in the proportion of children reporting fever, diarrhoea or vomiting. However, there were notable differences with reported cough. While both groups showed a reduction in children reporting cough after 14 days of follow-up, the proportion of children with cough was significantly lower in the RUTF group than in the control group (18.2% vs 37.2%; p = 0.006; Table 7). Additionally, while a similar proportion of children in both groups reported a reduced appetite at study inclusion, a significantly smaller proportion from the RUTF group reported reduced appetite at day 14. After 28 days, there was no difference in reported cough and appetite between the RUTF and control groups (Table 7).

## Discussion

Our findings indicate that 14 days of RUTF supplementation in children with malaria promotes increased rate of weight gain and a shorter period of convalescence, as shown by a significantly higher proportion of children reporting recovery from cough and improved appetite amongst children receiving RUTF supplementation. After 14 days, the RUTF group gained, on average, more weight and at a faster rate than children not receiving supplement, with the control group gaining close to the 1.4 g/kg/day expected amongst well children [15]. With the greater proportion of children gaining any weight in the RUTF group during the first 14 days, these results suggest a shorter convalescence period, reducing the period that a child is in a vulnerable state.

Contributing to this vulnerability is the depletion of nutritional elements, including proteins, essential fatty acids and essential micronutrients (incl. zinc, vitamins C, E vitamins and B), during active infection. This can decrease immunity and complicate recovery [16]. Infections, including malaria, are associated with anorexia and weight loss. With marginal micronutrient stores, two causes of disease-related anorexia and weight loss arise: i.) infection dependent regulation of appetite and ii.) secondary micronutrient deficiency.

Firstly, an infection-activated immune system increases cytokine production, particularly tumour necrosis factor (TNF) which is associated with anorexia. In the absence of micronutrients, essential for the regulation of the immune system, anorexia can persist unchecked.

Secondly, a highly active immune system increase the use of micronutrients, including the micronutrients (zinc, selenium) that, when deficient, are associated with decreased appetite and stunting. A supplementation of micronutrients, proteins and energy adds extra nutrients to mitigate the secondary deficiencies and promote weight gain. This is further supported by results showing a greater weight increase amongst children who reported low appetite at inclusion.

Similar to the results on weight gain, cough was significantly reduced at day 14 in the RUTF group compared with the control group. However, after 28 days, cough was equally reduced in both groups. These results suggest that food supplementation promotes a shorter convalescence with recovery of cough in children with malaria, giving hope that this approach could be effective amongst children with other diseases. Research shows also that micronutrient supplementation plays an important role in recovery from disease (eg, zinc contributes to the cure of and is protective for diarrhoea and pneumonia).

In settings of poor food security, one could expect to see even greater weight gain than that seen in this study. The relatively stable food security in the Dubie region might have enabled caretakers to provide adequate, or even additional, food for the children recovering from malaria. This likely contributed to a smaller positive impact of RUTF, given that the baseline nutritional state of a child will influence the effect of supplementation. At the time of the study, active agriculture was occurring and the overall food security situation in Dubié was fairly stable. In addition, an observed low prevalence of moderate malnutrition in children screened for study inclusion indicates a fairly good overall nutritional situation during the study period.

As an effect modifier, the slower rate of weight gain seen among partially breastfed children may be explained, in part, by the fact that breastfeeding limits weight loss during illness, thus reducing the amount of weight needed to be gained to attain pre-illness weight. A study by Brown *et al*. amongst sick children showed that breast-milk intake was close to normal, while intake of food (not breast milk) was significantly reduced amongst similar (non-breastfed) children [17]. In light of this and the risk that supplementation could disrupt healthy breastfeeding practices, further discussion and research is warranted as to the role of supplementation amongst breastfed children.

While the mean weight gain itself is likely not of clinical importance, for children with an already low weight and marginal nutrition state, such small improvements in weight gain and shorter convalescence are important for healthy recovery, decreased vulnerability, and decreased risk of malnutrition. Supplementation breaks the vicious cycle of malnutrition and disease; firstly by preventing or rapidly mitigating nutritional depletion during recovery and secondly by shortening the convalescence period after infection. While this study highlights the possible benefits, larger studies are needed to further confirm that supplementation to ill children can be a key contributor to reducing incidence of malnutrition and disease.

### Limitations

Mean weight gain is likely not the optimum outcome measure for measuring the effect of a supplementation on nutritional health. The incidence of malnutrition and of morbidity would more accurately measure the effectiveness of supplementation in breaking the cycle of malnutrition and disease amongst sick children.

With neither patient nor clinician blinded to group allocation, there is the risk of reporting and treatment bias. Monitoring of adherence to supplementation through caretaker interview and counting empty sachets at day-14 cannot definitively determine if the product was consumed by the targeted child, for example. Teams worked to keep such limitations to a minimum through standard health messages during clinic visits. However, the intention of this effectiveness study was to measure the impact of supplementation in a typical OPD setting, where such limitations are difficult or impossible to control.

### Conclusions

Children receiving ready-to-use therapeutic food for 14 days after an uncomplicated episode of malaria had a faster weight gain and recovery from illness than children not given supplementation, reducing the period that children were at risk of malnutrition.

Although these findings are not adequately robust to justify general distribution of RUTF supplementation to a wider population (i.e. children using outpatient services with confirmed malaria and not severely malnourished) in settings similar to Dubié, additional food supplementation with RUTF for sick children is a likely scenario in precarious settings with inadequate food security or a high prevalence of malnutrition.

The use of RUTF warrants further investigation, since these results cannot yet be generalised to situations with different levels of food security or extrapolated to other diseases. While giving indication to the positive effect of supplementation with RUTF on malnutrition and disease, the study does not give precise answers on which patient subgroups (e.g. identified by age, nutritional status, or illness) might benefit most from such supplementation. Further study is needed in order to investigate whether food supplementation to sick children can lower the incidence of malnutrition and disease in a context with a lower nutritional status.

For reasons of cost-effectiveness, we need to investigate whether multi-micronutrient supplementation alone would have had a similar effect as RUTF. If nutritional supplementation is proven to be effective, either food or micronutrients alone should be considered as an additional treatment for children with serious diseases such as lower respiratory tract infection, malaria, and diarrhoea.

## Supporting Information

Checklist S1 CONSORT 2010 checklist.
(DOC)

Protocol S1 Trial protocol.
(DOC)
