# Supplementary material for: Ready-to-Use Therapeutic Food for Catch-Up Growth in Children after an Episode of Plasmodium falciparum Malaria: An Open Randomised Controlled Trial
Source: PLoS One. 2012 Apr 25;7(4):e35006. doi: 10.1371/journal.pone.0035006 (PMC3338820; doi:10.1371/journal.pone.0035006)
Supplement: Protocol S1 — Trial protocol. (DOC) [file pone.0035006.s002.doc]

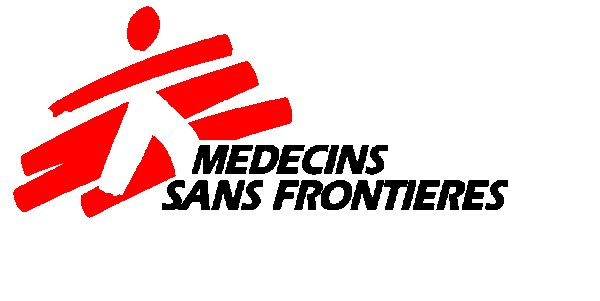


RESEARCH PROTOCOL

**The effectiveness of Ready to Use Therapeutic Food (RUTF) in catch up growth in children after an episode of *P. falciparum* malaria**

Democratic Republic of Congo (DRC)

Kilwa health zone, Katanga Province.

**Glossary**

DRC Democratic Republic of Congo

MIP Médecin Inspecteur Provincial

MSF-OCA Médecins Sans Frontières – Operational Centre Amsterdam

Plumpynut® A type of Ready To Use Therapeutic Food

RDI Recommended Daily Intake

RUTF Ready to Use Therapeutic Food

TFP Therapeutic Feeding Program

WHO World Health Organisation

**A randomised intervention study to evaluate the effectiveness of RUTF for catch up growth in children younger than 5 five years after an episode of malaria caused by *Plasmodium falciparum* in Dubie, Katanga, DRC.**

**Principle Investigator:**

Saskia van der Kam, MSF-OCA, Plantage Middenlaan 14, PO Box 10014, 1001 EA Amsterdam, The Netherlands. Email: saskia.vdkam@amsterdam.msf.org

**Research team:**

S. van der Kam^1^, A. Froud^2^, T. Swarthout^3^, O. Niragira ^4^, E. Mukomena Sompwe^5^, C. Mills^6^

*Specific scientific advisor: D. Klarkowski^7^ K. Ritmeijer*^2^*, K. Lokuge*^3^*, V. Lelubre*^4^

*Affiliations:*

^1^*= Principle Investigator, MSF-OCA*

^2^ *= Researcher MSF-OCA*

^3^ *= Epidemiologist MSF-OCA*

^4^ *=Medical Coordinator, MSF-OCA South DRC, Katanga*

^5^*= Chef de Division Provinciale de Santé/Province du Katanga or Médecin Inspecteur Provincial (MIP).*

^6^ *= Medical director MSF-OCA*

*^7^ = Laboratory specialist*

**Study Location:**

Dubie, Katanga, South DRC

**Objective:**

The primary objective of this study is to determine to what extent provision with RUTF will promote catch up growth in children following an acute uncomplicated episode of *P.* *falciparum* malaria.

**Method:**

Randomised intervention study.

**Number of patients:**

100 patients provided with 92 g (500 kcal) Plumpynut® (a Ready To Use Therapeutic Food (RUTF)) for 14 days, and 100 patients not provided with RUTF.

**Inclusion:**

Children aged 6 – 59 months with *P. falciparum* malaria, confirmed by thick film microscopy

**Exclusion:**

- - Children who are exclusively breast fed
  - Children who are severely malnourished
  - Presence of severe malaria

**Main outcome:** Average weight gain at 2 weeks post-enrolment in the group supplemented with RUTF, compared to the group not supplemented.

**Secondary outcomes:**

- Proportion of children not gaining weight in both groups
- Average weight gain at two weeks from the last day of supplementation.
- Contribution of other factors (potential confounders such as duration of malaria before treatment) to convalescence after an episode of malaria.

**A randomised intervention study to evaluate the effectiveness of RUTF for catch up growth in children younger than 5 five years after an episode of *P.falciparum* malaria in Dubie, Katanga, DRC.**

# 1. INTRODUCTION

**1.1 The inter-relationship between infection and malnutrition**

The aetiology of malnutrition in most tropical countries is multi-factorial, not only involving an inadequate diet but also recurrent infections.[[1]](#endnote-2) Specifically young children have a high exposure to acute infectious diseases (diarrhoea, acute respiratory infection) and they are at high risk of developing malnutrition. In many countries the situation seems to have worsened in recent years.[[2]](#endnote-3)

Infections are generally associated with weight loss during the illness. This occurs because infections may 1) decrease food intake (anorexia); 2) impair nutrient absorption; 3) cause direct nutrient losses; 4) increase metabolic requirements or catabolic losses; and 5) impair transport of nutrients to target tissues. Fever can account for 20-30% of reduced intake.[[3]](#endnote-4) In addition infections can cause increased losses (malabsorption, diarrhoea) and increased utilisation of nutrients (increased secretion in the gut, internal diversion for metabolic responses, increased metabolic rate when fever is present).[[4]](#endnote-5) As increased needs are not compensated by increased consumption, weight loss during an infection is frequent.[[5]](#endnote-6)

The weight loss occurring during an infection is not equal for all infections. Diarrhoea is known to induce weight loss and studies show that also malaria can result in a large weight loss. [[6]](#endnote-7), [[7]](#endnote-8) Lack of convalescence (i.e. failure to return to normal nutritional status) after an infectious disease makes a child more susceptible to further infections such as diarrhoea and malaria that, in turn, will reduce the nutritional state further. ^6,7^,[[8]](#endnote-9), [[9]](#endnote-10)

Effects of short-term weight loss during episodes of acute infection can be mitigated by good nutrition allowing for catch up growth.[[10]](#endnote-11),[[11]](#endnote-12) However, recovery of nutritional status is often not complete as good nutrition may be lacking. As a consequence a permanently lower nutritional status is established.[[12]](#endnote-13)

**1.2 Prevention of malnutrition**

Catch up growth after an infection is essential for an individual to maintain good nutritional status. In order to prevent malnutrition after an infection the World Health Organisation (WHO) recommends that children are given an extra healthy meal daily, during the two weeks after the onset of the illness.[[13]](#endnote-14) Studies on the effectiveness or impact of this strategy have never been published. However, in many poor regions this strategy is unlikely to be effective, as ingredients of a good meal are not always available and mothers/caregivers often lack resources and time to implement the advice. Therefore a more effective strategy in poor areas may be to provide a child with an infection a high quality supplement, rich in macro- and micronutrients, on contact with the health service. However, there is little evidence to support the effectiveness of this approach, apart from a study done in Gambia.^10^ In this study it was found that weight loss after acute diarrhoea was corrected by providing a supplement enriched with vitamins and minerals for 2 weeks after an episode of diarrhoea, while during non-intervention periods no significant weight gain occurred. This raises the question to what extent supplementation would be effective for infections other than diarrhoea, such as malaria.

**1.3 Relevance of the study**

Worldwide, malaria kills over a million people and each year 3.2 billion people are at risk. 80% of malaria deaths occur in Africa, where around 66% of the population is at risk.[[14]](#endnote-15) Country wide in the Democratic Republic of Congo (DRC), malaria is responsible for 68% of outpatient visits and 30% of hospital admissions, and accounted for 25-30 % of child mortality in DRC in 2005. Katanga Province had 640 000 reported cases of malaria in 2005 according to WHO statistics.[[15]](#endnote-16) In 2007 the clinics in Katanga supported by MSF-OCA in Katanga saw 54,671 children under five years, of whom 21,939 (40%) were treated for malaria.[[16]](#endnote-17)

The burden of malaria associated with malnutrition is illustrated by the admission profile in the Therapeutic Feeding Centre (TFC) in Dubie, where on average, 44% of the children admitted had/have a positive Paracheck® (February 2007).

Malaria is thus a huge burden on children in Katanga, which additionally puts them at risk of weight loss and poor convalescence ^6, 7^,[[17]](#endnote-18)

Given the significance of malaria in this setting, this study aims to explore the effectiveness of nutritional supplementation after an episode of malaria in preventing malnutrition. Other studies are also needed to explore the effect of supplementation after episodes of other serious diseases, such as pneumonia and severe diarrhoea.

If shown to be effective, MSF will consider incorporating routine nutritional support in its protocols as supportive therapy of serious infections, such as diarrhoea, malaria and pneumonia as a part of a strategy to prevent of malnutrition.

# 2. HYPOTHESIS

The primary objective of this study is to determine to what extent short-term provision with RUTF (Plumpynut®) will promote growth in children who have been diagnosed and treated for uncomplicated *P. falciparum* malaria. Therefore the following hypothesis will be tested:

Children aged 6-59 months presenting with malaria caused by *P. falciparum* who are provided with a RUTF supplement (Plumpynut®) of 500 kcal/day for 2 weeks will show significantly better catch up growth compared to a similar patient group not provided with RUTF (at 2 weeks and 4 weeks post-intervention).

Specific objectives of this study are:

- To assess the extent of lack of growth associated with an uncomplicated *P. falciparum* malaria episode in children 6-59 months who are not nutritionally supported
- To assess the extent the provision of RUTF for 2 weeks promotes weight gain during and after an uncomplicated *P. falciparum* malaria episode in children 6-59 months, at the end of the 2 weeks of supplementation.
- To assess the extent the provision of RUTF for 2 weeks promotes a sustained weight gain (as measured from presentation with *P. falciparum* malaria to 4 weeks after presentation).
- To assess to what extent other factors contribute to weight change after an episode of non-complicated acute *P. falciparum* malaria.

# 3. STUDY DESIGN AND METHODS

**3.1 Study design**

The study design is that of a randomised intervention study. Children with confirmed *P. falciparum* malaria, meeting all study inclusion criteria, will be enrolled in the study for a period of 28 days. Participants will be assigned at random to either the intervention or the control group.

The intervention group will receive treatment for malaria plus 1 bag of RUTF per day for 14 days. The control group will receive treatment for malaria without provision of RUTF.

Random allocation to either intervention or control group is thought to be acceptable by the population as RUTF is seen as a medicine rather than a food item. A similar study did not meet a problem with randomisation.[[18]](#footnote-2) For other illnesses, the participants will be offered medical treatment according to the best local standards.

Follow-up visits will be scheduled for directly observed treatment for malaria (day 0, 1 and 2), malaria parasite density and clearance (D0, D2, and until slide is negative; and for weight measurements at day 0, 1, 13 and 27. Weight will be measured twice at each visit by two independent observers. A detailed questionnaire will be conducted at the start, and at day 13 and 27.

All participants who attend follow up sessions at the clinic will receive a small incentive[[19]](#footnote-3)* in order to compensate for lost time due to participation in the study (RUTF will not be given to the control group at the end of the study).

The study will be conducted in the period (May-June) that is likely to have a stable prevalence of malnutrition, a stable incidence of *P. falciparum* malaria and other morbidities (e.g. pneumonia and diarrhoea). The total duration of the data collection will be 2 months for data gathering: 4 weeks of enrolment (40 participants per week) and 4 weeks of follow up (the last follow-up being done 4 weeks after the last enrolment).

**3.2. Study population**

The population of study will consist of children aged 6 to 59 months diagnosed with uncomplicated *P. falciparum* malaria consulting the MSF-supported clinics in Dubie, Katanga, South DRC, and whose parent or guardian gives informed consent for study inclusion (see Annex 1).

The children will be selected from villages in close proximity to the clinics (not further than 30 minutes walking distance (approximately 5 kilometres), to minimise loss to follow-up.

**3.2.1 Inclusion criteria**

- Age 6 to 59 months, and
- Positive rapid diagnostic test (Paracheck®) and
- Thick smear showing infection with *P.* *falciparum* and
- Informed consent from parents or guardian aged at least 18 years.

**3.2.2 Exclusion criteria**

- Children who are exclusively breast fed or
- Children who are severely malnourished (MUAC <110 mm and/or bilateral oedema, or WHO weight-for-Height criteria <3 Z-scores) or
- Presence of general danger signs or signs of severe malaria as defined by the WHO criteria, or
- Known history of allergy to malaria drugs, or
- Having a sibling enrolled in the study.

**3.2.3 Withdrawal:**

- When one of the exclusion criteria develop during enrolment
- Protocol violation: failure to complete the directly observed malaria treatment
- Protocol violation: consuming less than 10 sachets of RUF in 2 weeks when in treatment arm
- Erroneous inclusion of the patient.

**3.2.4 Follow-up**

Compensation for returning to the clinic will be provided to encourage follow-up at day 14 and day 28, in both study groups. The value of the compensation (in kind) will be approximately equivalent to one-day labour costs as this is roughly equivalent to the travel time and time spent in the clinic.

Active follow-up will be attempted for those who do not attended their appointments.

**3.3. Sampling frame**

The nature of the intervention means that blinding of health workers and participants is not possible. In order to address observer bias, the interviews will be carried out by field staff who are not part of the normal health services staff of the study clinics.

The sample size is based on the hypothesis that, compared to a control group of children who receive routine malaria treatment without nutritional supplementation, children receiving supplementation will show significantly greater average weight gain over the study period.

The following is the basic formula for calculating required sample size in each group (assuming equal numbers sampled in each group) for the difference between two population means[[20]](#footnote-4) :

n>2 ((z 2α σ)/d)^2^

Where:

n=number sampled in each population

d= difference in sample means

σ =standard deviation

Z2α = standardised normal deviate exceeded by specified probability

Assumptions for sample size calculations have been based on two previous studies from the Gambia assessing the impact of infection on weight gain. The first study, carried out in 1977, demonstrated that the incidence of either malaria or diarrhoea have a significant negative effect on weight gain.^6^ The second study, an intervention study published in 1996, demonstrated that short-term nutritional supplementation for diarrhoea had a significant impact on weight gain.^10^ This study provides the parameters for the sample size calculation, while the first study provides the basis on which we extrapolate the evidence that nutritional supplementation may address post-infection lack of weight gain related to diarrhoeal disease, to a similar impact on post-malaria weight gain. The primary sample size calculation is based on demonstrating a statistically significant difference in mean weight gain/loss between the intervention and control groups at two weeks.

# Expected mean change in weight for control and intervention groups are: ^10^

- Control group 0.09 kg/14 days (standard deviation 0.44)

- Intervention group 0.44 kg/14 days (standard deviation 0.33)

(For all calculations, power of 80% and 5% level of significance for a two- sided test of hypothesis was used. The Z2α would be in this case1.96)

Applying the parameters on the formula given above for means of 0.09 and 0.44, and standard deviation of 0.44, results in a required sample size is 13. This is quite small because even though the standard deviation is comparatively large, the magnitude of the 2 means is very different.

However, this assumes equal variances in the two population means. Statistical software (STATA version 10.0, STATAcorp, College Station, Texas, USA, 2007), was used for the sample size calculation, which adjusts for unequal variances, and therefore gives a larger sample size of 20 per group.

Estimated sample size required per study group:[[21]](#footnote-5)*

- Sample size per group: 20
- Required samples size per group (adjusted for 20% loss to follow-up): **24**

However, the difference between our study and the 1996 study is that the subject of that study was weight gain after diarrhoea, while our study will assess weight loss/gain after an episode of malaria. The 1977 study on the relationship between disease and weight loss showed that both diseases are associated with weight loss, but that for diarrhoea weight loss was more prevalent than for malaria. As the sample size above is considered the minimum, we aim to increase the sample size in order to increase the power of the study to take into account the expected higher variation in weight loss of children with malaria compared to children with diarrhoea.

The total sample size is defined by the minimum sample size (see above) and a sample size that is defined by the number of subjects that can realistically be enrolled in a day during a period of four weeks; enrolment of 10 malaria patients per day for 4 weeks (5 working days per week) gives a sample size of 100 per study group. As not all children will be eligible due to the inclusion/exclusion criteria, a sample size of 80 is thought to be a realistic target.

The number of children presenting with malaria in the clinic outweighs the number to be enrolled: in May 2007, 600 children under 5 years with P. falciparum malaria were treated in 4 weeks. Therefore sampling 1 out of 3 malaria cases would result in a sample size of approximately 100 per study group.

Therefore the proposed sample size is 100 per group.

The sample will be drawn from the group of children who come to the clinic, have been clinically reviewed and are suspected malaria cases. If the malaria rapid diagnostic test is positive, from this group **one in three** child will be selected randomly, and invited to join the study. This selection procedure is necessary as more children will be presenting with malaria then study can process in a day. After explaining the study and the consent procedure, the patient will be randomly allocated to either the control group or the intervention group. The random number function of a pocket calculator will be used for randomisation. In the first step numbers ending on 3,6 and 9 will be invited for the study, numbers ending on 1,2,4,5,7,8, will not be invited, and when a number ends no 0 a new random number will be generated. Numbers ending on an even number or a zero will be in the intervention group, numbers ending on uneven will be in the control group.

**3.4 RUTF supplement**

### RUTF item

RUTF is designed for catch up growth in malnourished children and therefore RUTF is also expected to promote catch up growth and weight gain after a period of anorexia. In this study, Plumpynut® will be the RUTF provided. Of the RUTF’s available, Plumpynut® is the most widely accepted and it has proven effects on child growth.[[22]](#endnote-19), [[23]](#endnote-20), [[24]](#endnote-21)

### Quantity

The 1996 supplementation study provided a fortified food ad libitum. The average consumption was 117 kcal/kg/day, and the average study subject weight was between 6.8 and 7.7 kg; this means a total supplement of 796 to 900 kcal/day was given. ^10^

MSF has chosen to provide a fixed quantity supplement, as currently packaged and used in existing programmes. This contains 500 kcal/day. Although this supplement has a lower energy value than the study in Gambia, it is liked by children and of very high quality. In addition, the energy (calories) provided is not the only criteria for supplementation, as lack of catch up growth also depends on the availability of micronutrients. The supplement must provide at least 1 RDI (Recommended Daily Intake) of the anti oxidant vitamins and minerals important for the production of erythrocytes (Vitamin A, B, C, selenium, zinc, folic acid, copper, iron). One package of Plumpynut® provides about 1 RDI (see annex 6).

# 4. CASE DEFINITION AND MAIN OUTCOME MEASURES

**4.1 Diagnosis**:

Malaria caused by *P. falciparum* will be diagnosed using a step- wise approach:

1. Screening for clinical signs of malaria
2. Rapid test (Paracheck®)
3. Laboratory confirmation.

During the initial screening of children presenting at the clinic, those with fever >38 degrees and/or a history of fever within the past 2 days will have a rapid test (Paracheck®) performed.[[25]](#endnote-22) Other symptoms that might be related to a malaria infection (e.g. anaemia, splenomegaly, headache) can indicate a Paracheck® to confirm malaria. Cases with a negative Paracheck® will be excluded. In order to exclude false positive Paracheck® test results (the test may remain positive for up to 6 weeks), a positive Paracheck® will be followed by laboratory confirmation (thick blood film). Those with no parasitaemia will be excluded.

In children with a positive blood film for *P. falciparum,* clinical signs will be assessed to exclude severe (complicated) malaria. If anaemia is clinically suspected the haemoglobin will be measured to exclude severe anaemia. Those children with a positive Paracheck®, positive blood film for *P. falciparum* and no other exclusion criteria will be eligible for the study and their caregivers will be asked for consent to enrol.

**4.2 Outcome parameters:**

**Main outcome:**

Average weight gain (g/kg/day) in the intervention group from day 1 to day 14 will be compared with the average weight gain in the control group.

**Secondary outcomes:**

- Proportion of children having lost weight in each group
- Sustained weight gain compared to short term weight gain (as assessed at 2 weeks after completion of the supplementation period (i.e. 4 weeks post intervention)
- Contributing factors for weight change (see details of potential confounders/effect modifiers in the following section).

# 5. CONFOUNDING FACTORS

There are many factors potentially influencing weight gain or loss other than a given supplement. These confounders may influence the study outcome. In reviewing previous literature, the following known confounders for convalescence^15^ or for the relationship between malaria and nutritional status^6^, ^9^, [[26]](#endnote-23) will be measured:

- Duration of the complaints before attending the clinic [[27]](#endnote-24)
- Duration of symptoms of malaria before attending the clinic
- Age of child (effect modifier ^23^); as age may be unreliable length/height can be used as a matched variable
- Breastfeeding (partial, as exclusive breastfed children are excluded)
- Disease history (e.g., respiratory tract infections, diarrhoea, vomiting, anorexia) during the last 2 weeks before enrolment
- Incident diseases during the study period
- Socio-economic status of the family
- Educational status of the caretaker and head of the household
- Maternal age
- Nutritional status at baseline
- Being resident or displaced.

# 6. DATA COLLECTION

**6.1 Admission**

On admission, a questionnaire will be administered (see annex 3), and anthropometry, medical examination, and lab tests performed:

- Demographics: Name, age, address
- Anthropometry: height, weight, MUAC, oedema
- Medical status: fever, danger signs for severe malaria, loss of appetite, concomitant illnesses
- Laboratory data: RDT result, thick blood film and level of parasitaemia
- Medical history: duration of symptoms, breastfeeding (exclusive or partial), appetite
- Socio-economic: main source of income, whether receiving food aid, number of meals consumed.

**6.2 Day 14 after treatment**:

At day 14 a further questionnaire (see annex 4) will be administered, in addition to anthropometry measurements and repeat medical examination:

- Anthropometry: weight, MUAC, oedema
- Medical status: fever, appetite, intercurrent illnesses,
- Laboratory data: thick blood film and level of parasitaemia
- Medical history: duration of symptoms, new illnesses.

**6.3 Day 28:**

At day 28 a further questionnaire (see annex 4) will be administered, in addition to anthropometry measurements and repeat medical examination:

- Anthropometry: weight, MUAC, oedema
- Medical status: fever, appetite, intercurrent illnesses
- Medical history: new illnesses.

# 7. QUALITY CONTROL

**7.1 General:**

An additional expatriate research supervisor will be assigned for 3,5 months to give training, and supervise and monitor the implementation of the study. Clinical procedures and data collection and will be evaluated regularly by the research supervisor to ensure that the protocol is being followed correctly. Patient records will be double checked on a random basis. A random sample of interviews and clinical procedures will be observed, and the Paracheck® procedure will be evaluated according to a standard checklist.

**7.2. Staff, Training, and Supervision**

There will be 4 additional staff for 2 months to ensure adequate assessments at admission, discharge and follow-up of defaulters, for monitoring during the treatment phase and for performing the laboratory work. As the staff will not be blinded for selection of participants or for allocation of the supplement the potential exists for observer bias. This will be addressed by selecting staff- to be responsible for the interviews- who are not currently involved in health care provision within MSF clinics or having regular duties in the clinic.

Training of identified staff on the aims and procedures of the study and role of staff will take place before the study starts by the expatriate field research supervisor. Staff will be trained including practical demonstration of their specific tasks, such as interviews, weight measurements, and laboratory techniques (including the correct use of Paracheck®)

**7.3 Weight changes**

Weight gain is defined as the difference in weight between weight on day one and weight on day 14. The weight of an individual varies during the day and therefore ideally weight should be measured at the same time of the day on each follow up visit. As patients may initially present at any hour during the day, their enrolment in the study and the first weight taken will be at any time on this day (day 0). In order to standardise the time of taking weight, to limit intra-day variation, patients will be asked to come back for follow up within a certain period in the day (pre or post meal times). The follow up visits will be scheduled at the same period of the day. The weight at day 1 (the day after enrolment) will be used as baseline.

Weight changes in a short period are very small. Therefore even a small degree of measurement error could influence outcomes. By weighing each individual twice with two independent observers, measurement error will be calculated and the average will be taken for the analysis. Electronic weighing scales will be used (SECA 834 and 835) that have a precision of >=20g.

**7.4 Malaria treatment**

## In order to achieve a similar compliance with malaria treatment in the two study arms, directly observed malaria treatment will be implemented in both groups.

## Children will remain under observation for 30 minutes after treatment to ensure they don’t vomit their medication. If they do vomit the medication within 30 minutes, the same treatment regimen will be given again and children will again remain under observation for 30 minutes.

Parasite clearance will be monitored by blood slide readings on D2. Treatment failure will be assessed on D2, including parasitaemia higher than D0 count. In the case of treatment failure, a 2nd line treatment regimen will be used, as per local MSF treatment protocols. These children will be withdrawn from the study.

**7.5 Laboratory examination and quality control**

Extra training will be given to the laboratory staff about the rationale and methods of the study. MSF standard procedures for laboratory quality control will be applied. Routinely a thick blood film will be taken at D0 (after the result of the paracheck**®** test is positive) D2 and D13.

Blood films will be examined to determine the presence and the density of falciparum parasites. Slide results will be recorded in ‘number of parasites per microlitre (#/µL)’ and further be rated :+ =1-10 parasites per 100 thick film fields

++ =11-100 parasites per 100 thick film fields

+++ =1-10 parasites per single thick film field

++++ = more than 10 parasites per single thick film field

During the first 3 days of the study 20% of blood slides will be reread by a second microscopist. All weak positive slides will be crosschecked. Discrepant results will be assessed and clarified amongst the two available microscopists. All blood slides will be appropriately stored for future external QC; normal MSF quality control procedures will be implemented: 10% of the slides will be referred to a reference lab (PathCare in Nairobi) for cross-checking. This sample of 10% should show 100% agreement. If not, then all slides will be crosschecked.

Haemoglobin levels will only be assessed when anaemia is clinically suspected, as is the current clinical procedure.

# 8. DURATION OF THE STUDY

Training of the staff and pre-testing of the questionnaire (e.g. nurse, measurers, active case finding, laboratory staff) will take 1 week. Participants will be enrolled in 4 weeks (total one month). Follow up will extend for one month after enrolment of the last participant. The final analysis and writing up of reports will take 2 months.

# 9. INVESTIGATORS AND THEIR RESPONSIBILITIES

**9.1. Responsibilities**

The Public Health Department of MSF-OCA is responsible for the study. Only on the authority of the Medical Director or the MoH of DRC should the study be substantially altered or stopped. The principal investigator (PI), S van der Kam, is accountable to MSF-OCA.

A scientific advisory committee will advise the PI at crucial stages on design and implementation of the study.and includes:

- The principal investigator
- Members of the Public Health department at HQ levels knowledgeable in research or extensive knowledge of the area;
- The medical responsible in Katanga for MSF-OCA programs (medco) is responsible for input in the research design on issues related to local circumstances, overall organization of the study and all communications with the authorities concerning the study.
- The field research supervisor will be responsible for communication with stakeholders in the community and ensuring the quality of diagnosis, treatment, the completeness of case report forms, follow-up visits and data entry. who is the responsible for field implementation.
- The Médecin Inspecteur Provincial of Katanga is responsible for the government’s commitment to the study, and suggestions to make the study appropriate for the population concerned.
- The Medical Director of MSF-OCA is the final overall responsible;

The research team who are directly involved in the execution of the research consists of the Principle investigator, the medical responsible for MSF–OCA programs in Katanga, the field research supervisor and the medical director MSF OCA.

**9.2. Authorship:** Authors will be drawn from the study group and will be considered to be **authors** if they have made a substantial personal contribution (in time and effort) to the design, implementation, analysis, and write-up of the study. Other study group members will join the list of **contributors** that will be acknowledged in any publications or presentations.

# 10. RECORDS, DATA PROCESSING & ANALYSIS/REPORTING

**Patient records**

Patient records specific to the study will be kept on the study site and handled confidentially, and will be kept at MSF offices at capital level after follow up for storage. All data (paper records and electronic data) will remain the property of MSF-OCA.

As patients approach the clinic for medical care, standard procedures for patient care will be followed and patient medical cards will remain in possession of the patient (as per current standard procedures in the Dubie health services).

**Data entry**

Data will be entered twice in EpiData/Excel, and analysed in Epi-Info and SPSS.

**Data analysis**

The primary analysis will be aimed at determining the impact of the intervention on mean weight change over the study period. The impact of the intervention will be assessed using multivariate linear regression, with intervention status included as an explanatory variable in a model adjusted for all major confounders identified in previous studies and found to be significant in the primary analysis.

Secondary analyses will include assessing the proportion of children with weight gain post malaria and the impact of intervention on this, and evaluation of factors associated with weight gain at 4 weeks compared to 2 weeks post-intervention.

**Reporting**

The PI will be responsible for drafting the report of this study in collaboration with the epidemiologist of MSF HQ. Publication in an appropriate medical scientific journal will be sought. The PI is responsible for ensuring the collaboration and consent of all co-workers, including the epidemiologist and for submitting an agreed version of the final report within three months of completion of the study.

# 11. ETHICAL CONSIDERATIONS

Ethical considerations are fundamental to the design of any research involving human beings, and have been taken into consideration throughout the design of this study.

**Cooperation with national and local partners** in Katanga Province, DRC:

For this study, cooperation is established with the National Health Authorities in Kilwa (Médecin Chef de Zone) and the Province (Médecin Inspecteur Provincial of Katanga), who is a member of the study group. MSF-OCA feels this cooperation is essential for accessing and providing information to the key decision makers involved in the definition of effective measures to combat malnutrition. The local administrative authorities will be consulted about the nature and time-schedule of the planned study.

The details of the study and its implications will be explained to health staff in the clinics in the area. As the study involves a temporary benefit (RUTF) for the treatment of the study group it is essential that community members understand the purpose of the investigation and agree with the procedures. MSF-OCA field visitors will inform village chiefs and community members (specifically women with young children).

**Benefits to the community**: Based on the outcome of this study MSF will consider adapting protocols to include provision of RUTF for children with serious acute illness. If shown to be effective, this would contribute to improving survival of children in the community, as assumed is that improved nutrition in general will improve survival. The study also hopes to contribute to an improved understanding in the community of the necessity for good nutrition after an acute infection. The opportunity will be taken to instruct as many doctors and nurses as possible in the importance of good nutrition after an infection and the impact on growth of disease.

**Potential risks:** Plumpynut® is safe for children with malaria. Although research has suggested that iron supplementation during an episode of malaria can negatively influence the outcome of the disease[[28]](#endnote-25), the iron content in Plumpynut® (5 mg iron /sachet) is not considered as a major problem, as it is much lower than normally used therapeutic doses (3 mg/kg/day). Food based delivery of iron has substantially different patterns of absorption and metabolism compared to iron supplementation through pills. [[29]](#endnote-26) In addition, in this study, malaria will be treated immediately upon admission, and thus any potential effect of iron in the food on the parasite will be very limited.

**Scientific merit:** There are substantial gaps in knowledge and evidence relating to the effectiveness of providing nutritional support on acute illness and convalescence. There is an urgent need to investigate whether a short nutritional intervention can achieve complete nutritional recovery. The design and conduct of this pilot study aims to start to answer some of those evidence gaps in a robust way in a field setting.

**Informed consent:** Parents/guardians of the patients will be informed about the purpose of the study, the procedures when participating in the study, and asked for their consent. Informed consent forms will be translated into local languages and back-translated; they will be piloted for comprehension in the field. As a significant proportion of the population is not literate, the consent form will be read out to the patient’s parent/guardian to obtain oral consent as required (see annex 1).

It will be made clear that participation is voluntary and that patients will receive the same standard of medical treatment for malaria whether or not they agree to participate in the study. Patients can withdraw from the study when they wish at any point.

**Confidentiality:** The medical examinations and the interviews will be performed in private (a separate room) to ensure patient confidentiality. Patient data will be only accessible to the investigators and not given to any unauthorized person. In further data processing, patient data will be anonymous, and only given study sequential numbers.

Optimal medical treatment:

Decisions regarding the management of patients will be based on their clinical condition, independent of the study. Participation in the study will not, at anytime, preclude supplementary treatment or a change in therapy if clinically indicated. The well-being of the patient will always be a priority over participation in the study. Directly observed treatment for malaria medication is chosen to ensure optimal compliance with treatment.

**Feedback of results:** The individual results of weight change will be shared and discussed with the caretaker. At the end of the study, feedback and preliminary outcomes will be provided to the stakeholders in the community and to medical authorities.

## Follow up of children with a disease who are excluded from the study

- Illnesses in children with or without malaria presenting to the clinic will be treated according to the best standards available to MSF in that community.
- Children with severe malnutrition will be referred to the existing MSF Therapeutic Feeding Program (TFP) to be treated adequately. The 2005 WHO growth standards will be applied, ensuring that all children at risk for mortality related to malnutrition will be selected for therapeutic feeding.

**Independent review:** The Ethical Review Board of MSF will review the study protocol. Up to now there has been no appropriate ethical review committee identified in Katanga. Permission from local authorities in the districts of study will be obtained.

## ANNEX 1: Informed Consent

*[To be read in French or local language(s), whichever the patient requests. Thereafter,* if literate***,*** *caretakers will be each given a printed copy of this sheet and allowed to study the paper and reflect on it at least 15 minutes. Patients will then individually be asked for consent to participate and a record of their consent made]*

Dear parent/guardian:

Sick children often do not eat well and can lose weight. This can lead to malnutrition. MSF, in collaboration with the Ministry of Health, want to test whether giving children a special food will help them gain weight after being treated for malaria. We will compare the weight changes of children who eat this special food with those not eating it.

Your child has tested positive for malaria and is younger than five years and older than six months. Therefore we want to ask you to participate in this study. If your child has signs of severe malaria, is severely malnourished, is allergic for malaria drugs, is exclusively breastfed or is having a sibling in the study already, your child won’t be included in the study.

If you agree, your child will either receive treatment for malaria alone, or your child will receive malaria treatment plus a special food (Plumpynut®). You will be allocated to one of treatments by chance (like in a lottery). You cannot choose which group your child will be allocated to and neither can the health staff.

If you agree to participate, we will ask you some questions about the health and feeding of your child and some about your own background. The treatment will be started today but we want you to return to the clinic tomorrow and the following day so we can see your child take the medicine and measure his/her weight. Also we want to ask you to return again after 2 and 4 weeks for checking your child’s weight and to ask you some questions. If you agree to be in the study, it is important that your child returns to the clinic for these planned visits even if he/she is not sick.

If your child does not show up on the scheduled appointment, we’d like to know if it is ok if a member of the MSF staff visits you at home to remind you to come to the clinic. If you think that you will not be able to bring the child back, let us know now. The child will still receive treatment as usual, but he/she cannot be included in the study.

At any time during the study, even outside the schedule of appointments, if the child feels sick you should bring him/her back for a check up, even on weekends. It doesn’t cost anything.

You don’t have to agree to let your child participate – it’s up to you to decide, and whether or not you agree to participate, there will be the same care offered to your child. Also, you may stop bringing the child to the study at any time and for any reason, without any further consequences for you or your child and future treatment in the clinic.

Participating will not cost you or your family anything. You will receive soap and a jerry can for coming back to the health post for weighing the child and answering the questions.

The information we collect will not be shared with any others besides the health staff involved with the care of your child. The information used for the study will not have your child’s name on it.

Do you have any questions about the study? Please ask all the questions that you feel like asking. If you accept to participate, please confirm that you are willing to do so through the procedure (below).

**Consent form:**

*The caretaker may wish to give her/his consent verbally, or wish to sign her/his consent form – either is acceptable. In case of oral consent the text below should be read out in French or the local language. In addition a member of the staff should record that the information was understood and the consent freely given.*

I, (name)................ ……………………………………………………………………..

of (address) ........…………………………………………………aged ......…………..years,

Caretaker of:

Name of child ………………… ……………………….

Give permission for the child’s participation in the study comparing weight gain after treatment for malaria with or without additional nutrition support. The purpose of the study, the way it is to be conducted, and the inconveniences and risks that may reasonably be expected have been explained to me. I have been given the opportunity to ask questions concerning this study, and these questions were answered to my satisfaction. Should I have any further questions concerning this study, I may ask the nurses treating my child. I understand that I can withdraw from the study or decide to stop my child’s treatment at any stage without having to give a reason, and that MSF staff will continue to look after me to their best ability.

Caretaker signature

AND/OR

Witness name: ............................…………………….

Witness signature: ............................…………………….

Date: ................

Any queries pertaining to this study can be addressed to the Field supervisor (*Name and contact details*) or Principal Investigator:

Saskia van der Kam

MSF-Holland

Max Euweplein 40

P.O.Box 10014

1001 EA Amsterdam

The Netherlands

tel: (+31) 20 520 8978

fax: (+31) 20 620 5170

e-mail: Saskia.vd.kam@amsterdam.msf.org

| Annex 2: RUTF malaria recovery study | | | | | | | | | | | | |
| --- | --- | --- | --- | --- | --- | --- | --- | --- | --- | --- | --- | --- |
| Checklist for inclusion/exclusion | | | | | | | | | | | | |
|  | | | |  | |  | | |  |  | |  |
| Paracheck number | | | |  | |  | | |  |  | |  |
| Paracheck positive: | | | | Yes |  | **🡪 Continue** | | |  |  | |  |
|  | | | | No |  | **🡪 Do not enrol in the study** | | | | | | |
|  | | | |  | |  | | |  |  | |  |
| Child between 6 months and 5 years old? | | | | Yes |  | **🡪 Continue** | | |  |  | |  |
|  | | | | No |  | **🡪 Do not enrol in the study** | | | | | |  |
|  | | | |  | |  | | |  |  | |  |
| Date (dd/mm/yy): | | | |  | |  | | |  |  | |  |
|  |  | | |  | |  | | |  |  | |  |
| **A. Danger signs / severe malaria:** | | | | | | | | |  |  | |  |
|  | 1. Unable to drink/breastfeed | | | | | | | |  | Y | | N |
|  | 2. Vomiting everything | | | | |  | | |  | Y | | N |
|  | 3. Recent history of convulsions | | | | | | | |  | Y | | N |
|  | 4. Unable to sit or stand | | | | |  | | |  | Y | | N |
|  | 5. Any other signs of severe malaria (if yes, specify) | | | | | | | | | Y | | N |
|  |  | | |  | |  | | |  |  | |  |
|  | **Any of the questions above answered with Yes? 🡪 Do not enrol in the study and refer for immediate treatment** | | | | | | | | | | | |
|  |  | | |  | |  | | |  |  | |  |
| **B. Signs of anaemia** | | | | | | | | |  |  | |  |
|  | 1. Some or severe palmar pallor | | | | | |  | |  | Y | | N |
|  | 2. Mucous membrane pallor | | | | |  | | |  | Y | | N |
|  | 3. Respiratory distress | | | | |  | | |  | Y | | N |
|  |  | | | | | | | | |  | |  |
|  | **Any of the questions above yes? Test Hb; HB<5 g/dL? 🡪 Do not enrol in the study and offer normal treatment** | | | | | | | | | | |  |
|  |  | | |  | |  | | |  |  | |  |
| **C. Severe malnutrition** | | | | | | | | | | | | |
|  | 1. Bilateral oedema | | | | | | | Yes | | **No** |  | |
|  | 2. MUAC < 11 cm | | | | | | | Yes | | **No** |  | |
|  | 3. Weight-for-Height < -3sd WHO 2005 reference | | | | | | | Yes | | No |  | |
|  |  | | |  | |  | | |  |  | |  |
|  | **Any of the questions above answered with Yes? 🡪 Do not enrol in the study and refer for immediate treatment in the TFP** | | | | | | | | | | | |
|  |  | | |  | |  | | |  |  | |  |
| **D. Other criteria - please tick:** | | | | | |  | | |  |  | |  |
|  | 1. Serious concomitant illness | | | | |  | | |  | Y | | N |
|  | 2. History of allergy to anti malarial drugs | | | | | | | |  | Y | | N |
|  | 3. History of allergy to peanuts or milk | | | | | | | | | Y | | N |
|  | 4. Exclusive breastfeeding | | | | | | | | | Y | | N |
|  | 5. Sibling enrolled in this study | | | | | | | | | Y | | N |
|  | 6. Lives further away than 30 min walking distance | | | | | | | |  | Y | | N |
|  | (check name of village) | | | | | | | |  |  | |  |
|  | **Any of the questions above answered with Yes? 🡪 Stop** | | | | | | | | | | |  |
|  |  | | |  | |  | | |  |  | |  |
| **E. Consent:** | | | | | | | | |  |  | |  |
| *Explain the study and read out consent form* | | | | | | | | |  |  | |  |
|  | Guardian > 18 and close relative, gives consent | | | | | | | |  | Y | | N |
|  | |  | | | | | | |  |  | |  |
| Included? | Y | | N |  | | | | |  |  | |  |
|  |  | |  |  | | | | |  |  | |  |
| Study ID number |  | |  |  | | | | |  |  | |  |

# *Consent forms are presented in Annex 1.*

| Annex 3: RUTF for malaria recovery study |
| --- |
| **Intake questionnaire upon enrolment** |

| Date (dd/mm/yy): |  |  | | | |  | |  |  | |  | | | |  |
| --- | --- | --- | --- | --- | --- | --- | --- | --- | --- | --- | --- | --- | --- | --- | --- |
| Patietn ID |  |  | | | |  | |  |  | |  | | | |  |
|  |  |  | | | |  | |  |  | |  | | | |  |
|  |  |  | | | |  | |  |  | |  | | | |  |
|  |  |  | | | |  | |  |  | |  | | | |  |
| Sex | Male | | Female | | |  | |  |  | |  | | | |  |
|  |  |  | | | |  | |  |  | |  | | | |  |
| **Part 1: Questions concerning your child** | | | | | | | |  |  | |  | | | |  |
|  |  |  | | |  | | |  |  | |  | | | |  |
| 1. What is the exact age of your child? | | | | | | | |  |  | |  | | | |  |
|  |  | Birth date of child (dd/mm/yy) | | | | | |  |  | |  | | | |  |
|  | ***Or*** | Age of child (months, years). | | | | | |  |  | |  | | | |  |
|  |  |  | | |  | | |  |  | |  | | | |  |
| 2 You have brought your child because he/she is ill. How many days ago did this episode of illness start? | | | | | | | | …… | days | |  | | | |  |
|  |  |  | | |  | | |  |  | |  | | | |  |
|  | | | | | | | |  |  | |  | | | |  |
| 3. Has your child been ill with a fever at any time in the last 2 weeks? | | | | | | | | Yes |  | |  | | | |  |
|  |  |  | | |  | | | No |  | |  | | | |  |
|  |  |  | | |  | | | Don’t know |  | |  | | | |  |
|  |  |  | | |  | | |  |  | |  | | | |  |
|  |  |  | | |  | | |  |  | |  | | | |  |
| 4. Has your child had an illness with cough at any time in the last 2 weeks? | | | | | | | | Yes |  | | 🡪 Q 5 | | | |  |
|  |  |  | | |  | | | No |  | | 🡪 Q 7 | | | |  |
|  |  |  | | |  | | | Don’t know |  | | 🡪 Q 7 | | | |  |
|  |  |  | | |  | | |  |  | |  | | | |  |
|  |  |  | | |  | | |  |  | |  | | | |  |
| 5. When your child had an illness with a cough, did she/he breathe faster than | | | | | | | | Yes |  | | 🡪 Q 6 | | | |  |
| usual with short, rapid breaths or have difficulty breathing? | | | | | | | | No |  | | 🡪 Q 7 | | | |  |
|  |  |  | | |  | | | Don’t know |  | | 🡪 Q 7 | | | |  |
|  |  |  | | |  | | |  |  | |  | | | |  |
| 6. Was the fast or difficult breathing due to a problem in the chest or | | | | | | | | Chest only | | |  | | | |  |
| to a blocked or runny nose? | | | | | | | | Nose only | | |  | | | |  |
|  |  |  | | |  | | | Both | | |  | | | |  |
|  |  |  | | |  | | | Don’t know | | |  | | | |  |
|  |  |  | | |  | | | Other; specify | | |  | | | |  |
|  |  |  | | |  | | |  |  | |  | | | |  |
| 7. Has your child had diarrhoea in the last 2 weeks? | | | | | | | | Yes |  | |  | | | |  |
| *(Explain the definition of diarrhoea: at least 3 or more liquid stools per day )* | | | | | | | | No |  | |  | | | |  |
|  |  |  | | |  | | | Don’t know |  | |  | | | |  |
|  |  |  | | |  | | |  |  | |  | | | |  |
| 8. In the last 2 weeks, has your child vomited everything? | | | | | | | | Yes |  | |  | | | |  |
| (persistent vomiting several times) | | | | |  | | | No |  | |  | | | |  |
|  |  |  | | |  | | | Don’t know |  | |  | | | |  |
|  |  |  | | |  | | |  |  | |  | | | |  |
| 9. Do you currently breast-feed your child? | | | | | | | | Yes |  | | 🡪 Q 10 | | | |  |
|  | | | | | | | | No |  | | 🡪 Q 11 | | | |  |
|  | | | | | | | |  |  | |  | | | |  |
|  | | | | | | | |  |  | |  | | | |  |
| 10. How many times during the day did you breastfeed yesterday? | | | | | | | | …… times | | | | | |  |  |
|  | | | | | | | |  | | | | | |  |  |
| 11. **Since your child was ill**, did she/he eat (food or breast milk) | | | | | | | | Much less | | | | | |  |  |
| less then usual, about the same amount, or more than usual? | | | | | | | | Somewhat less | | | | | |  |  |
| IF LESS, PROBE: Was he/she eating much less than usual or somewhat less? | | | | | | | | About the same | | | | | |  |  |
|  | | | | | | | |  | | | | | |  |  |
|  | | | | | | | | Somewhat more | | | | | |  |  |
|  | | | | | | | | Much more | | | | | |  |  |
|  | | | | | | | | Don’t know | | | | | |  |  |
|  | | | | | | | |  |  | | |  | | |  |
|  |  |  | | |  | | |  |  | | |  | | |  |
| **Part 2: The following questions concern yourself or the entire family** | | | | | | | |  |  | | |  | | |  |
|  | | | | | | | |  |  | | |  | | |  |
|  | | | | | | | | Mother | | | |  | | |  |
| 12: What is your relation to the child? | | | | | | | | Father | | | |  | | |  |
|  | | | | | | | | Grandparent | | | |  | | |  |
|  | | | | | | | | Brother /sister | | | |  | | |  |
|  | | | | | | | | Other | | | |  | | |  |
| 13. What is your own age? (years) …………..years | | | | | | | |  | | | |  | | |  |
|  | | | | | | | |  | | | |  | | |  |
| 14. Where (in what village) do you currently live? ………………………………………….. | | | | | | | |  | | | |  | | |  |
|  | | | | | | | |  |  | | |  | | |  |
| 15.How long have you lived in Dubie or surroundings of Dubie? | | | | | | | | Always | |  | | 🡪 Q 17 | | |  |
|  | | | | | | | | …… …. | |  | | Years | | |  |
|  | | | | | | | |  |  | |  | | | |  |
| 16. Were you displaced because of the war? | | | | | | | | Yes | | | |  | | |  |
|  | | | | | | | | No | | | |  | | |  |
|  | | | | | | | | Don’t | know | | |  | | |  |
|  | | | | | | | |  |  | | |  | | |  |
| 17. What is the highest level of education that you have completed? | | | | | | | |  |  | | |  | | |  |
| *Please tick the box of the highest level of education* | | | | | | | |  |  | | |  | | |  |
|  |  | No formal schooling | | | | | |  |  | | |  | | |  |
|  |  | 1-4 years of primary school | | | | | |  |  | | |  | | |  |
|  |  | More than 4 years primary school (but not completed) | | | | | |  |  | | |  | | |  |
|  |  | Primary school completed | | | | | |  |  | | |  | | |  |
|  |  | 1-3 years Secondary school (but not completed) | | | | | |  |  | | |  | | |  |
|  |  | Secondary school completed | | | | | |  |  | | |  | | |  |
|  |  | High school | | | | | |  |  | | |  | | |  |
|  |  | College/pre-university/University (but not completed) | | | | | |  |  | | |  | | |  |
|  |  | College/pre-university/University completed | | | | | |  |  | | |  | | |  |
|  |  | Don’t know | | | | | |  |  | | |  | | |  |
|  | | | | | | | |  |  | | |  | | |  |
| 18. What is your current marital status? | | | | | | | |  | | | |  |  | |  |
|  |  |  | | | | | Single |  | 🡪 Q 21 | | | | | |  |
|  |  |  | | | | | Currently married |  | 🡪 Q 19 | | | | | |  |
|  |  |  | | | | | Cohabiting |  | 🡪 Q 19 | | | | | |  |
|  |  |  | | | | | Divorced/Separated |  | 🡪 Q 21 | | | | | |  |
|  |  |  | | | | | Widowed |  | 🡪 Q 21 | | | | | |  |
|  | | | | | | | |  | | | |  | | |  |
| 19. Is your husband/partner living with you now or is he staying elsewhere? | | | | | | | | Living with her/him | | | |  | | |  |
|  | | | | | | | | Staying elsewhere | | | |  | | |  |
|  | | | | | | | |  | | | |  | | |  |
| 20. What is your husbands’/ partners occupation. That is, what kind | | | | | | | | Agriculture | | | |  | | |  |
| of work does he mainly do? | | | | | | | | Trading/shop | | | |  | | |  |
|  | | | | | | | | Regular job | | | |  | | |  |
|  | | | | | | | | Other , specify | | | |  | | |  |
|  | | | | | | | |  | | | |  | | |  |
| 21. What is your occupation, that is, what kind of work do you mainly do? | | | | | | | | Agriculture | | | | 🡪 Q22 | | |  |
|  | | | | | | | | Trading/shop | | | | 🡪 Q23 | | |  |
|  | | | | | | | | Regular job | | | | 🡪 Q23 | | |  |
|  | | | | | | | | Other , specify | | | | 🡪 Q23 | | |  |
|  | | | | | | | |  |  | | |  | | |  |
| 22. Do you mainly work on your own land or on family land, or do you work | | | | | | | | Own land | | | |  | | |  |
| on land that you rent from someone else, or do you work on someone else’s land? | | | | | | | | Family land | | | |  | | |  |
|  | | | | | | | | Rented land | | | |  | | |  |
|  | | | | | | | | Someone else’s land | | | |  | | |  |
|  | | | | | | | |  | | | |  | | |  |
|  | | | | | | | |  |  | | |  | | |  |
| 23. How many hectares of agricultural land do members of this household own? | | | | | | | | None | | | |  | | |  |
| (in total) | | | | | | | | ............ | | | | Hectares | | |  |
| (Household definition: people sleeping in the same house and eating from the same cooking pot) | | | | | | | | Don’t know | | | |  | | |  |
|  | | | | | | | |  |  | | |  | | |  |
| 24. Does any member of your household own | | | |  | | | | Yes | No | | | Don’t know | | |  |
|  | | | | A watch | | | |  |  | | |  | | |  |
|  | | | | A bicycle | | | |  |  | | |  | | |  |
|  | | | | A radio | | | |  |  | | |  | | |  |
|  | | | | Have a generator for electricity | | | |  |  | | |  | | |  |
|  | | | | | | | |  |  | | |  | | |  |
|  | | | | | | | |  |  | | |  | | |  |
| 25. Does this household own any livestock, herds, other form animals, or poultry? | | | | | | | | Yes | | | | 🡪 Q26 | | |  |
|  | | | | | | | | No | | | | 🡪 Q27 | | |  |
|  | | | | | | | | Don’t | know | | | 🡪 Q27 | | |  |
|  | | | | | | | |  |  | | |  | | |  |
|  | | | | | | | |  |  | | |  | | |  |
| 26. How many of the following animals does this household own? | | | | | | | |  |  | | |  | | |  |
|  | | | |  | | | |  |  | | | Number | | |  |
|  | | | |  | | | | Goats | | | |  | | |  |
|  | | | |  | | | | Sheep | | | |  | | |  |
|  | | | |  | | | | Chicken / other poultry | | | |  | | |  |
|  | | | |  | | | | Pigs | | | |  | | |  |
|  | | | |  | | | | Cows | | | |  | | |  |
|  | | | | | | | |  |  | | |  | | |  |
| 27. What is the main material of the external walls of your house? | | | | | | | |  |  | | |  | | |  |
|  | | | |  | | | | Yes | No | | | Don’t know | | |  |
|  | | | | Improvised: plastic, cloth, timber | | | |  |  | | |  | | |  |
|  | | | | Wood, mud, bamboo | | | |  |  | | |  | | |  |
|  | | | | Stone, brick, cement | | | |  |  | | |  | | |  |
|  | | | | Don’t know | | | |  |  | | |  | | |  |
|  | | | |  | | | |  |  | | |  | | |  |
|  | | | | | | | |  |  | | |  | | |  |
|  | | | | | | | |  |  | | |  | | |  |
| 28. Did you get any food aid the last month? | | | | | | | | Yes |  | | |  | | |  |
|  |  |  | | |  | | | No |  | | |  | | |  |
|  |  |  | | |  | | | Don’t know |  | | |  | | |  |
|  |  |  | | |  | | |  |  | | |  | | |  |
| 29. How many meals did the family have yesterday | | | | | | | |  |  | | | meals | | |  |
|  |  |  | | |  | | |  |  | | |  | | |  |
|  |  |  | | |  | | |  |  | | |  | | |  |

| Annex 4 : RUTF for malaria recovery study |
| --- |
| **First and second follow up questionnaire (14 and 28 days)** |

| Study ID |  | | | |  | |  | | |  | | |
| --- | --- | --- | --- | --- | --- | --- | --- | --- | --- | --- | --- | --- |
| Sex | M | | F | |  | |  | | |  | | |
| Date (dd/mm/yy): |  | | | |  | |  |  | |  | | |
|  | | | | | | |  |  | |  | | |
| 1. Did the complaints of your child disappear in the last 2 weeks? | | | | | | |  |  | |  | | |
| If not, why do you think they didn’t? | | | | | | |  |  | |  | | |
|  | | | | | | |  |  | |  | | |
| 2. Has your child been ill with a **fever** at any time in the last 2 weeks? | | | | | | | Yes |  | |  | | |
|  | |  | |  | |  | No |  | |  | | |
|  | |  | |  | |  | Don’t know |  | |  | | |
|  | |  | |  | |  |  |  | |  | | |
| 3. Has your child had an illness with **cough** at any time in the last 2 weeks? | | | | | | | Yes |  | | 🡪 Q 4 | | |
|  | |  | |  | |  | No |  | | 🡪 Q 6 | | |
|  | |  | |  | |  | Don’t know |  | | 🡪 Q 6 | | |
|  | |  | |  | |  |  |  | |  | | |
| 4. When your child had an illness with a cough, did she/he breathe faster than | | | | | | | Yes |  | | 🡪 Q 5 | | |
| usual with short, rapid breaths or have difficulty breathing? | | | | | | | No |  | | 🡪 Q 6 | | |
|  | |  | |  | |  | Don’t know |  | | 🡪 Q 6 | | |
|  | |  | |  | |  |  |  | |  | | |
| 5. Was the fast or difficult breathing due to a problem in the chest or | | | | | | | Chest only | | |  | | |
| to a blocked or runny nose? | | | | | | | Nose only | | |  | | |
|  | |  | |  | |  | Both | | |  | | |
|  | |  | |  | |  | Don’t know | | |  | | |
|  | |  | |  | |  | Other; specify | | |  | | |
|  | |  | |  | |  |  |  | |  | | |
| 6. Has your child had **diarrhoea** in the last 2 weeks | | | | | | | Yes |  | |  | | |
| *(Explain the definition of diarrhoea: at least 3 or more liquid stools per day )* | | | | | | | No |  | |  | | |
|  | |  | |  | |  | Don’t know |  | |  | | |
|  | |  | |  | |  |  |  | |  | | |
| 7. In the last 2 weeks, has your child vomited everything? | | | | | | | Yes |  | |  | | |
| (persistent vomiting several times) | | | | | |  | No |  | |  | | |
|  | |  | |  | |  | Don’t know |  | |  | | |
|  | |  | |  | |  |  |  | |  | | |
| 8. Has your child visited a health facility since your previous visit? | | | | | | | Yes | |  | | |  |
|  | | | | | | | No | |  | | |  |
| 9. Which medication has your child taken since your previous visit? | | | | | | |  | |  | | |  |
| (other than the anti-malaria) | | | | | | | None | |  | | |  |
|  | | | | | | | Drug: | | | | |  |
|  | | | | | | |  | | | | |  |
| 10. Do you feel the child is completely recovered from the malaria episode? | | | | | | | Yes | | | | |  |
|  | | | | | | | No | | | | |  |
| If not, why do you think the child did not recover yet? | | | | | | | Don’t know | | | | |  |
|  | | | | | | |  | | | | |  |
|  | | | | | | |  | | | | |  |
|  | | | | | | |  | | | | |  |
| 11. **During the last 2 weeks** did she/he eat (food or breast milk)? | | | | | | | Much less | | | | |  |
| less then usual, about the same amount, or more than usual. | | | | | | | Somewhat less | | | | |  |
| *IF LESS, PROBE: Was he/she eating much less than usual or somewhat less* | | | | | | | About the same | | | | |  |
|  | | | | | | | More | | | | |  |
|  | | | | | | | Somewhat more | | | | |  |
|  | | | | | | | Much more | | | | |  |
|  | | | | | | | Don’t know | | | | |  |
|  | | | | | | |  | | | | |  |
|  | | | | | | |  | | | | |  |
|  | | | | | | |  | | | | |  |
|  | | | | | | |  | | | | |  |
| **ATTENTION: Question 12, 13 and 14 are only for the RUTF group on day 13** | | | | | | |  | | | | |  |
|  | | | | | | |  | | | | |  |
| 12. During the last 2 weeks how many days did your child eat 1 complete | | | | | | | ........ days | | | |  | |
| Plumpynut? | | | | | | |  | | | | |  |
|  | | | | | | |  | | | | |  |
| 13. How many plumpynut has your child eaten the last 14 days? | | | | | | |  | | | | |  |
|  | | | | | | |  | | | | |  |
| 14 How many empty sachets are returned? | | | | | | |  | | | | |  |
|  | | | | | | |  | | | | |  |

Thank you.

**Annex 5: Sources and justification of questions in questionnaires:**

References:

- Measure DHS Model Questionnaire; With Commentary. Basic Documentation. Number 2 ORC Macro, Calverton, Maryland’ July 2006[[30]](#endnote-27)

-World health survey, B – Individual Questionnaire Rotation – A, World Health Organization, Evidence and Information for Policy. 2002[[31]](#endnote-28)

Pre intake checklist (Annex 2):

A=annex; Q=question. B1: Sick child age 2 months up to 5 years WHO/EMRO chartbook[[32]](#endnote-29)

# Intake questionnaire upon enrolment (Annex 3):

A=annex; Q=question

A3Q2: “You have brought your child because he/she is ill. How many days ago did this episode of illness started?” Adapted from DHS Section 5 Q544: “How many days after the illness began did you first seek advice or treatment?”

A3Q3: DHS Section 5 Q533

A3Q4: DHS Section 5 Q534

A3Q5: DHS Section 5 Q535

A3Q6: DHS Section 5 Q536

A3Q7: DHS Section 5 Q518; case definition diarrhea; Source: Clinical Guidelines, MSF 2007

A3Q8: WHO ind Q6556

A3Q9: new question, in DHS breastfeeding is related to breastfeeding history from birth, therefore in the DHS there is no question about breastfeeding at the day of interview.

A3Q10. Adapted from DHS Congo-Brazzaville: “Hier, combien de fois avez-vous allaité pendant les heures de la journée?” The cut of point we will use for breastfeeding of importance is 2 times per day.

A3Q11: “Yesterday does she/he eat (food or breast milk) less then usual, about the same amount, or more than usual.” IF LES, PROBE: Was he/she eating much less than usual or somewhat less. Adapted from DHS Section 5 521. : “When (NAME) had diarrhea, was he/she given less than usual to eat, about the same amount, more than usual or nothing at all”. The DHS is measuring feeding behaviour during illness, while we want to know whether the child has eaten normally. Breastfeeding considered as food. The answer category of more is removed as it is not distinguishing between somewhat more and much more

A3Q17: WHO Q1009, answer categories are adapted to context

A3Q18 : WHO Q1008

A3Q19: DHS 604

A3Q20: DHS section 8 Q806, added answer categories

A3Q21: DHS section 8 Q811, added answer categories

A3Q22: DHS section 8 Q813,

A3Q23: DHS Household questionnaire 123 added definition of household

A3Q24: DHS Household questionnaire 111and 121 combined and adapted to local context

A3Q25: DHS Household questionnaire 124

A3Q26: DHS Household questionnaire 115, adapted to local context

A3Q27: DHS Household questionnaire 119, adapted to local context

A3Q28: indicator suggested by field team

A3Q29: Indicator used in nutritional surveys

# Follow up questionnaire (Annex 4):

A4Q2: DHS section 5 Q533

A4Q3: DHS Section 5 Q534

A4Q4: DHS Section 5 Q535

A4Q5: DHS Section 5 Q536

A4Q6: DHS Section 5 Q518; added case definition diarrhoea source…MSF WHO

A4Q7: WHO ind Q6556

A4Q8 and Q9: indicator suggested by epidemiologist

A4Q12,13,14 are added to measure plumpynut consumption

# ANNEX 6: Composition 1 ration Plumpynut® and Recommended Daily Intake

|  | ***Recommended Daily Intakes*** | | | ***Plumpynut***® |
| --- | --- | --- | --- | --- |
|  | **Child 7-12 months** | **Child 12-36 months** | **Child 4-6 years** | **per ration (92g)** |
| **Kcal** |  |  |  | 500 |
| **Vit A**  µg | 400 | 400 | 450 | 840 |
| **Vit D** µg | 5 | 5 | 5 | 15 |
| **Vit D3** µg |  |  |  |  |
| **vit E** mg | **2.7** | **5** | **5** | 18.4 |
| **vit B1** (Thiamin) mg | **0.3** | **0.5** | **0.6** | 0.55 |
| **vit B2** (Riboflavin) mg | **0.4** | **0.5** | **0.6** | 1.66 |
| **B6** mg | **0.3** | **0.5** | **0.6** | 0.55 |
| **Vit B12** µg | **0.7** | **0.9** | **1.2** | 1.7 |
| **Vit C** mg | **30** | **30** | **30** | 49 |
| **Folic Acid** µg | **80** | **150** | **200** | 193 |
| **Niacin** (B3) mg | **4** | **6** | **8** | 4.88 |
| **Vit K** µg | **10** | **15** | **20** | 19.3 |
| **Biotin** µg | **6** | **8** | **12** | 60 |
| **Pantoth.Acid** (B5) mg | **1.8** | **2** | **3** | 2.85 |
| **Calcium**  mg | **400** | **500** | **600** | 276 |
| **Phosphor** mg | *275* | ***460*** | ***500*** | 276 |
| **Potassium** mg | *700* | *3,000* | *3,800* | 1022 |
| **Magnesium** mg | **54** | **60** | **76** | 85 |
| **Zinc** mg | **0,8 - 8,4 ^3^** | **2,4 - 8,3** | **2,9 - 9,6** | 12.9 |
| **Copper** mg | *0.22* | *0.34* | *0.44* | 1.6 |
| **Sodium** mg | *370* | *1,000* | *1,200* | < 267 |
| **Iron** mg | **6,2 - 18,6** | **3,9 - 11,6** | **4,2 - 12,6** | 10.6 |
| **Iodin** µg | **90** | **90** | **90** | 92 |
| **Selenium** µg | **10** | **17** | **22** | 27.6 |
| **Manganese** mg | *0.6* | *1.2* | *1.5* | - |
| **Molybdenium** µg | *3* | ***17*** | ***22*** |  |
| **Chlorine** mg | *0.57* | *1.5* | *1.9* |  |
| **Chromium** µg | *5.5* | *11.0* | *15.0* |  |
| * in normal font: RNI from WHO-FAO 2004; recommended nutrient intakes (RNI) is the daily intake which meets the nutrient requirements of almost all (97,5%) apparently **healthy individuals** in an age- and sex-specific population  ** in ***italics bold font:*** RDAs (Recommanded Dietary Allowances), from Dietary Reference Intakes, US Institute of Medicine, Food and Nutrition Board, 2004 | | | | |
| *italics :* same source, AIs (Adequate intakes) | | | | |

**Annex 7: REFERENCES**

1. Goulet O, Lebenthal E, Branski D, Martin A, Antoine J, Jones PJH. Nutritional solutions to major health problems of preschool children: how to optimise growth en development. *J Pediatr Gastroenterol Nutr* 2006; S1-S3 [↑](#endnote-ref-2)
2. De Onis M, Frongillo EA, Blossner M. Is malnutrition declining? An analysis of changes in levels of child malnutrition since 1980. *Bull WHO* 2000 (78)1222-33 [↑](#endnote-ref-3)
3. Stephensen CB. Burden of infection on growth failure. *J Nut* S 1999; 534-538 [↑](#endnote-ref-4)
4. Bhutta, ZA, Effect of infections and environmental factors on growth and nutritional status in developing countries. *J Pediatr Gastroenterol Nutr* 2006; S13-S20 [↑](#endnote-ref-5)
5. Tomkins A, Watson F. Malnutrition and infection. A review. *Nutrition policy discussion paper No4*. ACCSCN 1989 [↑](#endnote-ref-6)
6. Rowland MGM, Cole TJ, Whitehead RG. A quantitative study into the role of infection in determining nutritional status in Gambian village children. *Br J Nutr* 1977 (37)411-450 [↑](#endnote-ref-7)
7. Shiff C, Checkley W, Winch P, Premij Z, Minjas J, Lubega P. Changes in weight gain and anaemia attributable to malaria in children living under holoendemic conditions. *Trans Royal Soc Trop Med Hyg* 1996 (90) 262-265 [↑](#endnote-ref-8)
8. Friedman FF, Kurtis JD, Mtalib R, Opollo M, Lanar DE, Duffy PE. Malaria is related to decreased nutritional status among male adolescents and adults in the setting of intense perennial transmission. *J Inf Dis* 2003 (188 ) 449-457 [↑](#endnote-ref-9)
9. Nyakeriga AM, Troye-Blomberg MT, Chemtai AK, Marh K. Williams TN. Malaria and nutritional status in children living on the coast of Kenya. *Am J Clin Nutr* 2004 (80)1604-1610 [↑](#endnote-ref-10)
10. Hoare S, Poppitt SD, Prentice AM, Weaver LT. Dietary supplementation and rapid catch-up growth after acute diarrhoea in childhood. *Br J Nutr* 1996 (76) 479-490 [↑](#endnote-ref-11)
11. Bahwere P, Mol de P, Donnen P, Dramaix-Wilmet M, Butzler JP, Hennart P, Levy J. Improvements in nutritional management as a determinant of reduced mortality from community-acquired lower respiratory tract infection in hospitalized children from rural Central Africa. Pead. Inf. Dis J. 2004 (8) [↑](#endnote-ref-12)
12. Tomkins AM. Protein-energy malnutrition and risk of infection. Proceedings of the Nutrition Society, Symposium on nutrition and resistance to infection. 1985 [↑](#endnote-ref-13)
13. Food, Water and Family Health, A manual for community educators. WHO 1994 [↑](#endnote-ref-14)
14. World Malaria Report. WHO 2005 [↑](#endnote-ref-15)
15. Country Profile Democratic Republic of the Congo. *Roll Back Malaria monitoring and evaluation*. DRC. WHO 2005 [↑](#endnote-ref-16)
16. MSF internal annual standard indicator report 2007. [↑](#endnote-ref-17)
17. Rowland MG, Rowland SG, Cole TJ. Impact of infection on the growth of children from 0 to 2 years in an urban West African community. *Am J Clin Nutr* 1988 (47)134-138 [↑](#endnote-ref-18)
18. Personal communication Dr. P. Ashorn, study is described at the website: http://www.clinicaltrials.gov/ct2/results?term=NCT00524446 [↑](#footnote-ref-2)
19. ^*^ Equivalent to a day labour costs, the exact item to be defined in the field [↑](#footnote-ref-3)
20. Reference: P. Armitage & G. Berry, Statistical Methods in Medical Research, Blackwell Scientific publications, Oxford 1994 [↑](#footnote-ref-4)
21. ^^ [↑](#footnote-ref-5)
22. Diop E ,Dossou NI, Ndour MM, Briend A, Wade S. Comparison of the efficacy of a solid ready-to-use food and a liquid, milk based diet for rehabilitation of severely malnourished children: A randomised trial. *Am J Clin Nutr 2*003 (78)302-307 [↑](#endnote-ref-19)
23. Linneman Z, Matilsky D, Ndekha M, Manary Micah J, Maleta K, Manary Mark J . A large-scale operational study of home-based therapy with ready-to-use therapeutic food in childhood malnutrition in Malawi. *Maternal & Child Nutrition* 2007 (3) 2006-215 [↑](#endnote-ref-20)
24. Defourny I, Seroux G, Abdelkader I, Harczi G. Management of moderate acute malnutrition with RUTF in Niger. *Field Exchange* (31) 2007 [↑](#endnote-ref-21)
25. MSF Clinical Guidelines 2007 [↑](#endnote-ref-22)
26. Friedman JF, Kwena AM, Mirel LB, Kariuki SK, Terlouw DJ, Phillips-Howard PA, Hawley WA, Nahlen BL, Shi YP, ter Kuile FO. Malaria and nutritional status among preschool children: results from cross-sectional surveys in Western Kenya. *Am J Trop Med Hyg* 2005 (73) 698-704 [↑](#endnote-ref-23)
27. Bhutta ZA, Effect of infections and environmental factors on growth and nutritional status in developing countries. *J Pediatr Gastroenterol Nutr* 2006; S13-S20 [↑](#endnote-ref-24)
28. Caulfield LE, Richard SA, Black RE. Undernutrition as an underlying cause of malaria morbidity and mortality in children less then five years old. Am J Trop Med Hyg 2004 (71) sup 2,55-63 [↑](#endnote-ref-25)
29. WHO. Iron supplementation of young children in regions where malaria transmission is intense and infectious disease highly prevalent. *WHO Statement* 2006 [↑](#endnote-ref-26)
30. Measure DHS, model questionnaire with commentary, basic documentation number 2. ORC Macro, Calverton Maryland. July 2006 [↑](#endnote-ref-27)
31. World health survey, B – Individual Questionnaire Rotation – A, World Health Organization, 2002 [↑](#endnote-ref-28)
32. Sick child age 2 months up to 5 years. WHO/EMRO chartbook 2005

    # ANNEX 7: activity scedule

    ***Descriptions of Activities Day-0 (screening)Day-1Day-2***

    ***Day-3Day-14Day-28***ScreeningXAnthropometry

    (weight, MUAC, oedema)XHx of diseaseXXXWeight / HeightXXXRapid diagnostic test (Paracheck)XBlood slideXXIf D2 slide not negativeMedical historyXXXTreatmentXXXRUTF given to intervention arm (14-days)XIntake formXFollow-up form

    (including any unplanned visit)XX [↑](#endnote-ref-29)
